# Ubiquitin-Modifying Enzymes as Cell-Fate Regulators in Intestinal Inflammation

**DOI:** 10.7150/ijbs.130460

**Published:** 2026-04-08

**Authors:** Chenchen Qian, Fangmin Ning, Yong Xu, Jingjing Shao, Binglu Shi, Chenjian Zhou, Yi Wang

**Affiliations:** 1 School of Pharmacy, Hangzhou Normal University, Hangzhou, Zhejiang 311121, China.; 2 Pharmacy Department, Wenzhou Central Hospital, Affiliated to Wenzhou Medical University, Wenzhou, Zhejiang 325000, China.

**Keywords:** cell fate, inflammatory bowel disease, ubiquitin-modifying enzymes, post-translational modifications

## Abstract

Inflammatory bowel disease (IBD), including ulcerative colitis (UC) and Crohn's disease (CD), is a chronic, relapsing inflammatory disorder of the gastrointestinal tract. Intestinal homeostasis relies on the intricate balance of cell fate decisions within the intestinal epithelium and immune compartments. Ubiquitin-modifying enzymes (UMEs), including E2 conjugating enzymes, E3 ubiquitin ligases, and deubiquitinating enzymes (DUBs), have emerged as pivotal molecular regulators of these processes by orchestrating post-translational modifications that dictate protein stability, activity, and localization. In this review, we systematically summarize the essential roles of UMEs in modulating diverse cell-fate outcomes and their subsequent effects on intestinal barrier integrity and immune responses. Furthermore, we discuss the pathogenic dysregulation of specific UMEs in IBD and highlight their potential as diagnostic biomarkers and therapeutic targets. Finally, we explore emerging strategies, including small-molecule inhibitors and PROTAC technology, for targeting UMEs in clinical applications. By integrating current advances, this review provides novel insights into the ubiquitin-mediated regulation of intestinal cell fate and offers new perspectives for the management of IBD and the prevention of colitis-associated cancer (CAC).

## Introduction

Inflammatory bowel disease (IBD), together with other forms of chronic colitis, represents a spectrum of persistent inflammatory disorders of the gastrointestinal tract, primarily including ulcerative colitis (UC) and Crohn's disease (CD) 1, 2. Its global incidence and prevalence have increased steadily, imposing a substantial healthcare burden 3. The pathogenesis of chronic colitis, including IBD, involves multiple interconnected mechanisms, including disruption of the intestinal epithelial barrier, endoplasmic reticulum (ER) stress, immune dysregulation, and intestinal microbiota dysbiosis 4, 5. Although agents targeting TNF-α and related pathways have been used in IBD treatment, their efficacy remains limited, with high recurrence rates and significant adverse effects 6. Therefore, identifying novel molecular mechanisms and therapeutic targets remains a major priority. Moreover, emerging evidence indicates that dysregulated cell fate programs represent a key pathological convergence point linking chronic intestinal inflammation and disease progression, including IBD and colitis-associated cancer (CAC) 7.

In this disease context, “cell fate” does not refer solely to transient changes in signaling activity but also encompasses irreversible or long-lasting biological outcomes that determine tissue integrity and immune equilibrium 8. These regulatory programs operate at both cellular and tissue levels. At the cellular level, they control epithelial cell survival or programmed cell death (including apoptosis, necroptosis, pyroptosis, and ferroptosis), intestinal stem cell renewal and differentiation, polarization and functional reprogramming of innate immune cells, and lineage commitment of adaptive immune cells 9-15. At the tissue level, these processes collectively shape pathological outcomes, including chronic inflammation, fibrotic remodeling, dysplasia, and CAC 16, 17. Collectively, cell fate regulation represents the terminal decision layer through which upstream inflammatory signals are translated into concrete cellular behaviors and disease-relevant pathological outcomes. Here, we emphasize that stress-adaptive responses, such as the unfolded protein response (UPR) and autophagy, are increasingly recognized as fate-modulating programs that influence cellular trajectories, rather than independent terminal fate endpoints.

Consistent with this framework, accumulating evidence indicates that ubiquitin-modifying enzymes (UMEs) act as key molecular switches linking inflammatory signaling networks to fate-determining programs. By controlling the stability, localization, and activity of core regulators involved in cell death, stress adaptation, differentiation, and immune activation, UMEs actively bias cellular trajectories toward survival versus death, repair versus injury, and immune tolerance versus chronic inflammation. Accordingly, ubiquitination and deubiquitination emerge as upstream determinants of cell fate decisions in the inflamed intestinal microenvironment.

Ubiquitin-modifying enzymes (UMEs), composed of ubiquitination enzymes (E1, E2, and E3 ligases) and deubiquitinating enzymes (DUBs), represent a central post-translational regulatory system governing protein homeostasis and signaling, thereby controlling protein fate and downstream cellular decisions 18, 19. Emerging evidence indicates that UMEs play critical roles in IBD pathogenesis by regulating epithelial cell survival and regeneration programs, immune cell activation and differentiation, and stress adaptation responses, thereby shaping epithelial barrier homeostasis and remodeling the inflammatory microenvironment 18-20. Several UMEs, such as A20, CYLD, OTULIN, and USP13, are aberrantly expressed in intestinal tissues from IBD patients and experimental models, and are closely associated with epithelial injury, immune dysregulation, and disease progression 21-25. However, systematic insights into the cell-type-specific functions, context-dependent roles, and dynamic regulation of UMEs in distinct IBD subtypes remain limited.

In this review, we systematically summarize recent advances in UME-mediated regulation of cell fate during intestinal inflammation. By integrating evidence across distinct cell types and signaling contexts, we highlight how these molecular switches orchestrate the transition between survival and death, repair and injury, and immune tolerance and chronic inflammation, thereby providing a conceptual framework for therapeutic targeting.

## Hierarchical regulation of UMEs in intestinal inflammation

### System overview, spatial organization, and chain-type specificity

**Figure 1:**
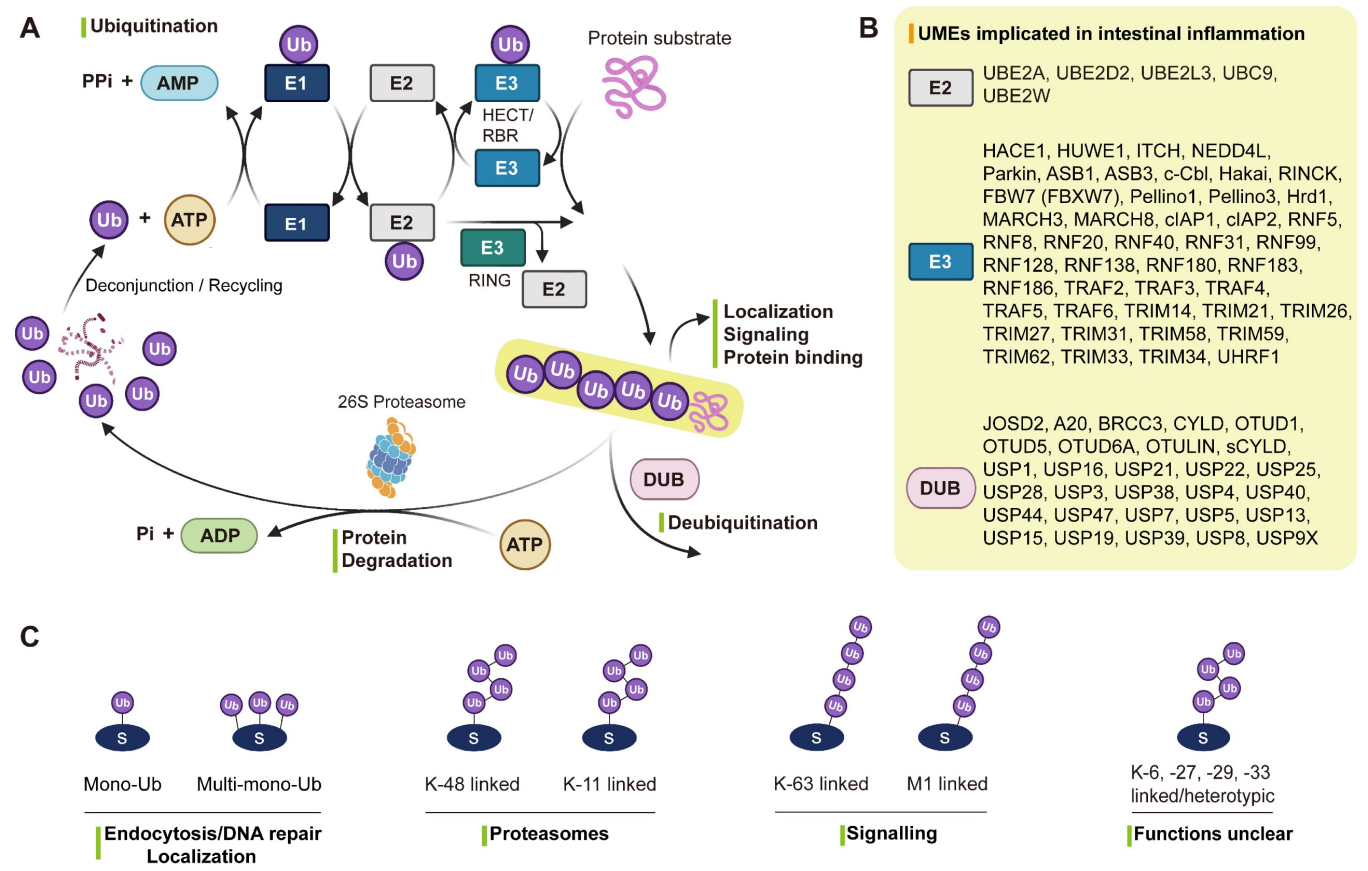
** Roles of UMEs in IBD. (A)** The ubiquitination cascade involves E1-mediated ubiquitin activation, E2-mediated transfer, and E3-catalyzed substrate modification, while DUBs remove ubiquitin and maintain signaling balance. **(B)** IBD susceptibility genes encode multiple UMEs, underscoring their critical roles in disease pathogenesis. **(C)** Distinct ubiquitin linkages (mono-, multi-mono-, and poly-ubiquitin chains such as K48, K63, and M1) confer specific cellular outcomes including degradation and signaling regulation. Created in BioRender. Qian, C. (2026) https://BioRender.com/u85g4fh.

The UME system comprises four enzymatic classes: E1 (activating), E2 (conjugating), E3 (ligating), and DUBs (removing) 26. This system enables precise regulation of protein stability, subcellular localization, interactions, and functional states, thereby coordinating diverse physiological and pathological processes 19, 27. During the enzymatic cascade, E1 activates ubiquitin (Ub) in an ATP-dependent manner, E2 transfers ubiquitin to E3, and E3 recognizes specific substrates to catalyze ubiquitin conjugation. Conversely, DUBs remove mono- or polyubiquitin chains from target proteins, ensuring the reversibility and dynamic regulation of ubiquitin signaling (**Figure 1A-B**).

Ubiquitin can be linked through seven lysine residues (K6, K11, K27, K29, K33, K48, K63) or the N-terminal methionine (M1) to form distinct chain types 28 (**Figure 1C**). Among them, K48/K11-linked chains mainly mediate proteasomal degradation 29, 30, while K63/M1-linked chains regulate inflammation and immune signaling 31. In contrast, mono-ubiquitination and atypical linkages such as K6, K27, K29, and K33 are involved in endocytosis, DNA repair, and vesicular trafficking 32. In IBD, the balance between different chain types and their removal by UMEs is finely tuned to control immune signaling, cell death, autophagy, and epithelial barrier integrity 33. Disruption of this dynamic equilibrium represents a central molecular mechanism driving chronic intestinal inflammation 33.

**Figure 2:**
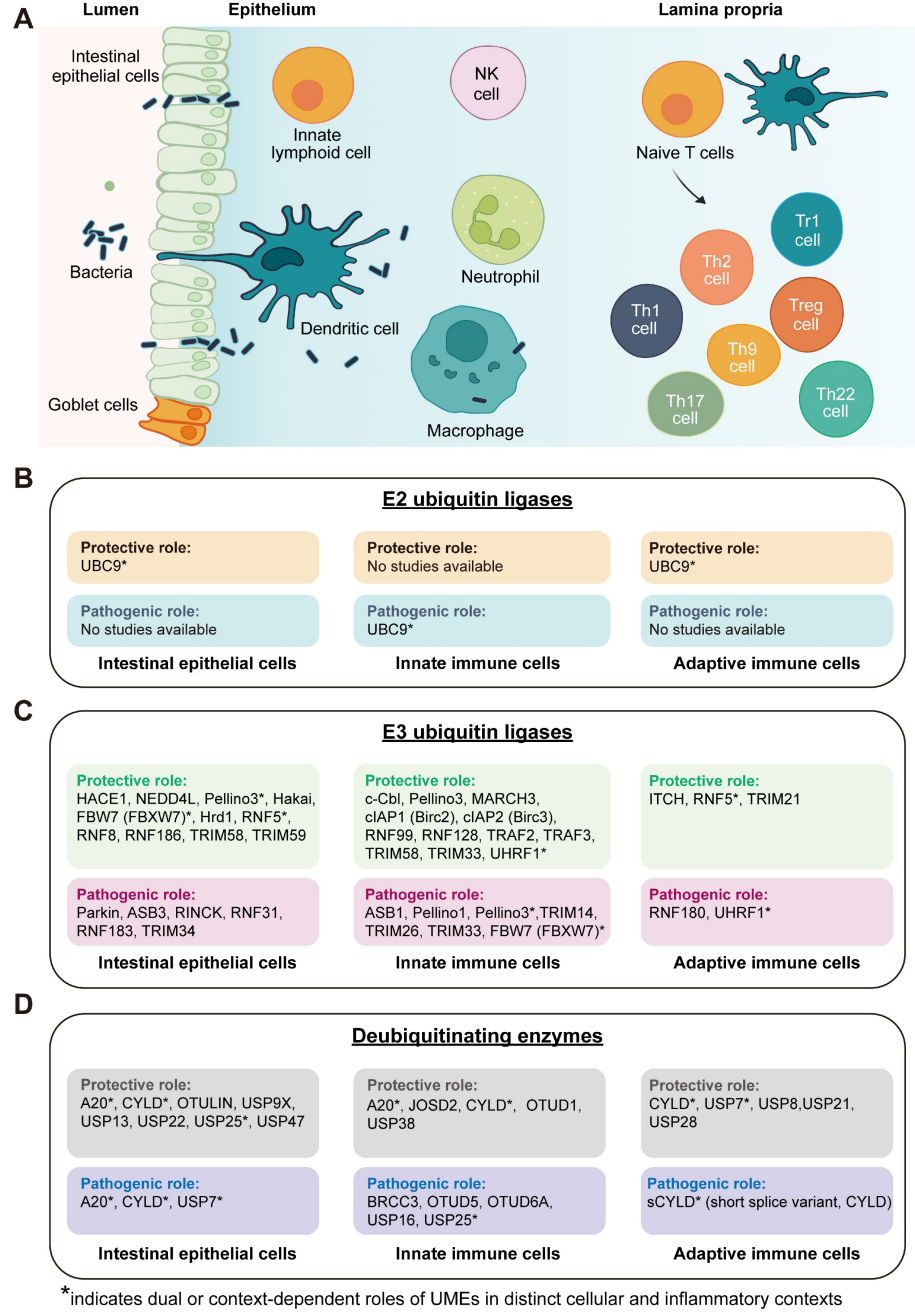
** Cell fate-layered landscape of UME functions in intestinal inflammation. (A)** Schematic overview of major cellular compartments involved in intestinal inflammation, including intestinal epithelial cells, innate immune cells, and adaptive immune cells across the epithelial barrier and lamina propria. **(B)** Summary of reported functions of E2 ubiquitin-conjugating enzymes across epithelial, innate immune, and adaptive immune compartments. **(C)** Classification of E3 ubiquitin ligases according to their reported protective or pathogenic roles in regulating epithelial and immune cell fate decisions. **(D)** Classification of DUBs based on their functional impact on inflammatory and stress-responsive pathways across distinct cellular compartments. Protective roles refer to functions that promote epithelial barrier integrity, immune homeostasis, resolution of inflammation, or tumor-suppressive outcomes, whereas pathogenic roles indicate activities that amplify inflammatory signaling, impair barrier function, or facilitate chronic inflammation and colitis-associated tumorigenesis. UMEs marked with asterisks (*) exhibit context-dependent or bidirectional effects depending on cell type, inflammatory stimulus, or disease stage. “Not yet studied” indicates the absence of direct experimental evidence in the corresponding cellular compartment rather than confirmed lack of function. Created in BioRender. Qian, C. (2026) https://BioRender.com/9xtir6c.

The intestinal immune system primarily comprises intestinal epithelial cells, innate immune cells, and adaptive immune cells (**Figure 2A**). Within this organized cellular landscape, UMEs display distinct cell type-specific distributions and functional roles **(Figure 2B-D)**, forming a multilayered regulatory architecture that coordinates epithelial barrier homeostasis and immune responses. This spatial and functional stratification provides a structural basis for the fine-tuned regulation of ubiquitin signaling during intestinal inflammation.

### Hierarchical regulation of cell fate by E2 ubiquitin-conjugating enzymes

**Figure 3:**
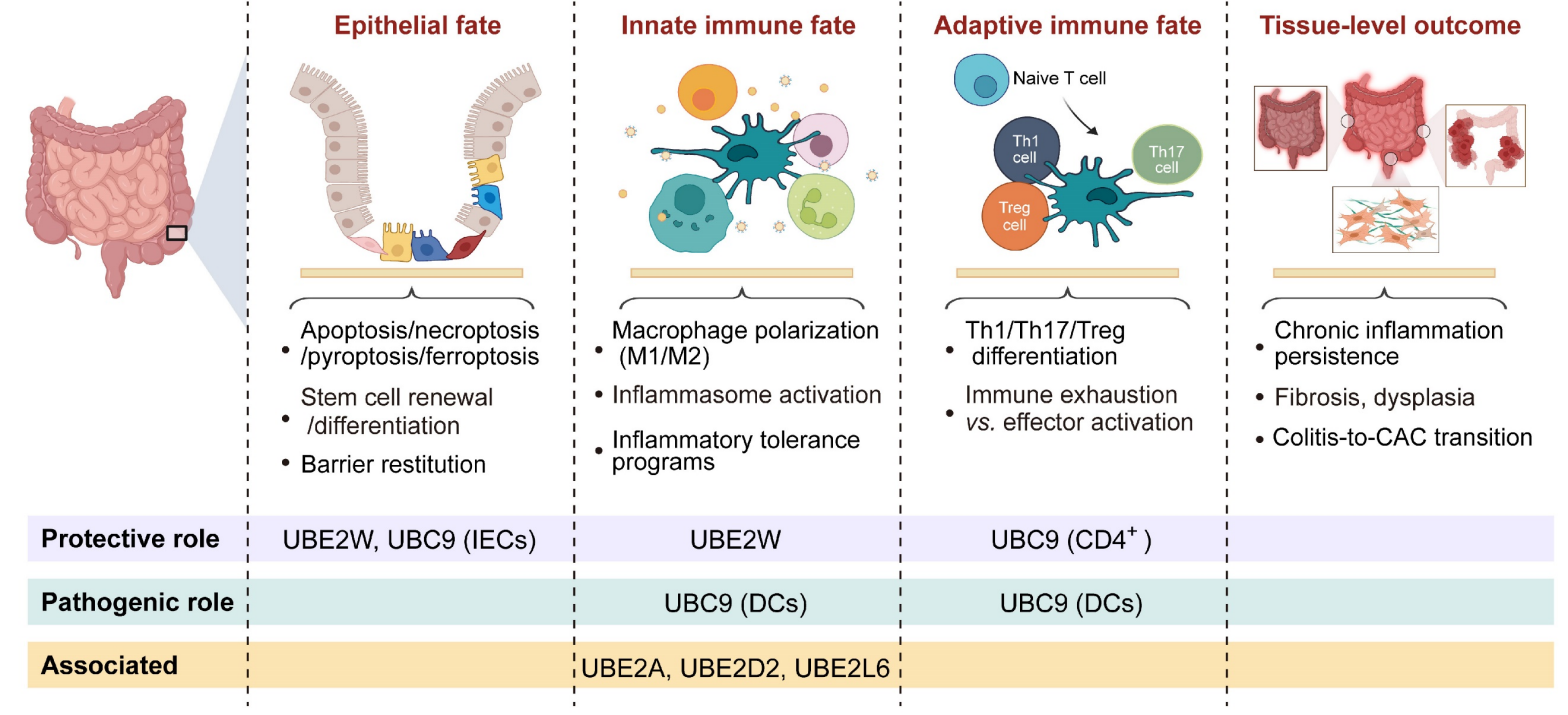
** Cell fate-layered organization of E2 ubiquitin-conjugating enzyme functions in intestinal inflammation.** Schematic illustrates the hierarchical organization of E2 enzyme-mediated regulation across interconnected cellular fate layers and downstream tissue-level outcomes during intestinal inflammation. Cellular fate layers include epithelial fate, innate immune fate, and adaptive immune fate, whereas tissue-level outcomes represent the integrated pathological consequences arising from the cumulative effects of these cellular programs. Representative biological processes regulated at each layer are indicated, including epithelial cell death and barrier restitution, macrophage polarization and inflammasome activation, T cell differentiation and immune exhaustion, as well as tissue-scale outcomes such as chronic inflammation and colitis-associated cancer (CAC) progression. Created in BioRender. Qian, C. (2026) https://BioRender.com/d0r43h0.

E2 ubiquitin-conjugating enzymes constitute the core module of the ubiquitination cascade, determining ubiquitin chain elongation modes, linkage specificity, and substrate fate 34. Beyond their enzymatic activity, accumulating evidence indicates that E2 enzymes also modulate substrate stability and signaling complex assembly, thereby regulating the amplitude and duration of inflammatory and stress-responsive pathways 35-38. In intestinal inflammation, E2-mediated regulation primarily tunes the amplitude of inflammatory signaling and the cellular activation thresholds, thereby influencing epithelial stress tolerance, antigen presentation, and immune polarization. Within the hierarchical cellular-to-tissue regulatory framework defined above **(Tables 1-2)**, E2 functions can be systematically mapped onto epithelial, innate immune, adaptive immune, and tissue outcome layers, highlighting their coordinated contributions to inflammatory progression and tissue remodeling **(Figure 2B, Figure 3, Table 3)**.

In intestinal inflammation, available evidence suggests that E2-mediated regulation primarily modulates the intensity of inflammatory signaling and the cellular activation thresholds. Gene expression analyses revealed that E2 enzymes, including UBE2A, UBE2D2, and UBE2L6, are differentially expressed in intestinal macrophages under inflammatory conditions in the gut 39. UBE2W attenuates DSS-induced colitis by limiting NF-κB-driven inflammatory amplification and epithelial barrier damage 40. UBC9 exhibits cell type-specific effects: in CD4⁺ T cells, reduced UBC9 enhances pathogenic Th17 responses 41; in IECs, UBC9 downregulation exacerbates NF-κB-dependent inflammation and colitis susceptibility 42; whereas in DCs, UBC9-mediated SUMOylation of RBPJ promotes T-cell activation and Th1/Th17 polarization, with DC-specific Ubc9 deletion alleviating experimental colitis 43. Collectively, these findings suggest that E2 enzymes act as critical regulators of intestinal immune homeostasis, influencing epithelial barrier integrity and immune cell polarization (**Figure 3, Table 3**). Notably, compared with the extensive characterization of E3 ligases and DUBs, current knowledge of E2 enzymes in intestinal inflammation remains limited and largely confined to a small subset of family members. Thus, E2-mediated regulation should be viewed as an emerging regulatory layer, with substantial gaps remaining in mechanistic and disease-level understanding.

### Hierarchical regulation of cell fate by E3 ligases

E3 ubiquitin ligases function as molecular switches that govern the stability and activity of key regulatory proteins during inflammation.

**Figure 4:**
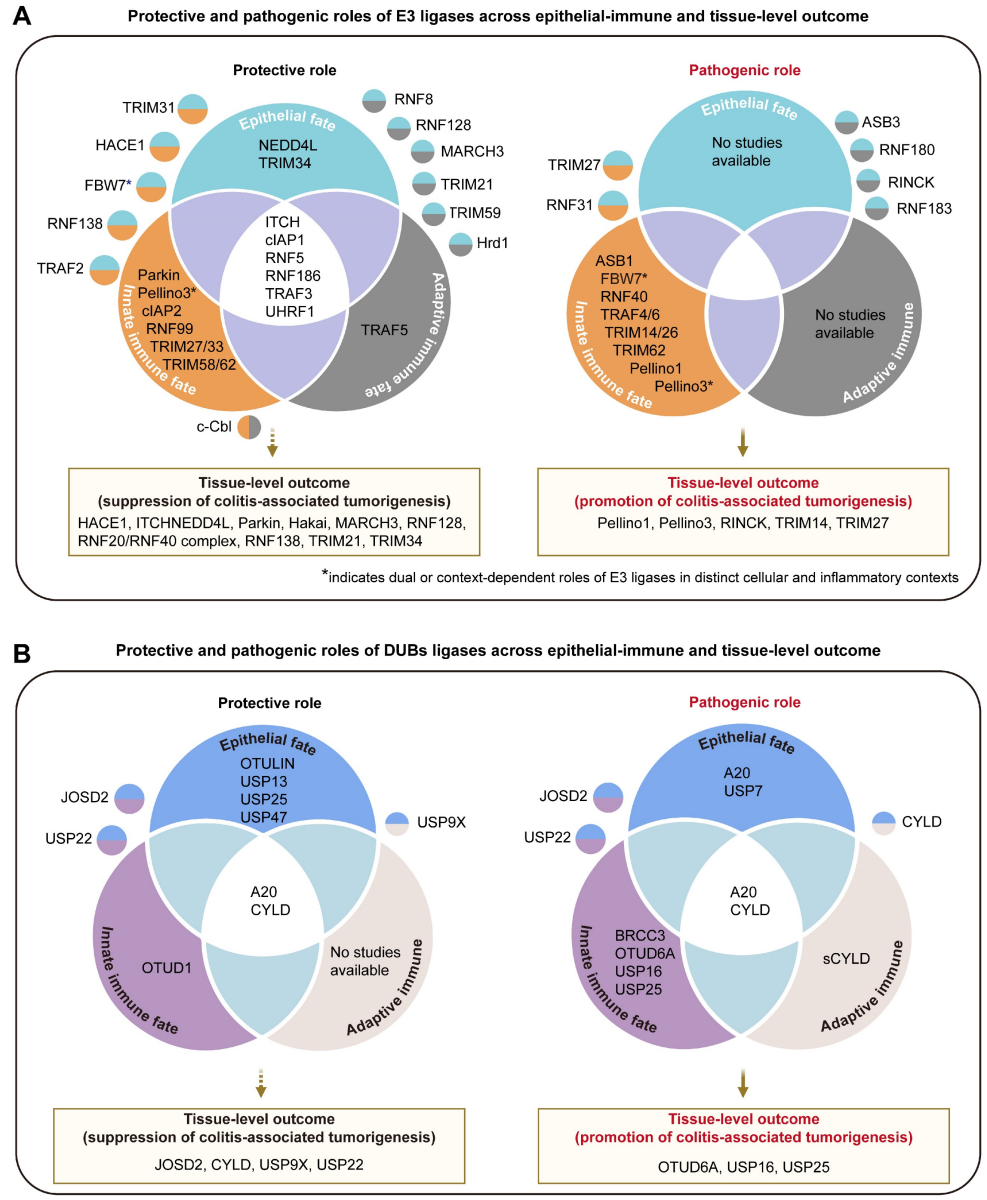
** Protective and pathogenic roles of UMEs in epithelial-immune cell fate layers and tissue-level outcomes during intestinal inflammation. (A)** Summary of E3 ligases and **(B)** deubiquitinases (DUBs) categorized according to reported protective or pathogenic functions across distinct cell fate layers, including epithelial, innate immune, and adaptive immune fate layers, together with their associated tissue-level outcomes related to colitis-associated cancer (CAC) progression. Venn diagrams illustrate UMEs that act within single compartments or coordinately regulate multiple cellular layers. UMEs shown in overlapping regions indicate shared regulatory roles across epithelial and immune compartments. Tissue-level fate panels summarize enzymes implicated in promoting malignant transition or suppressing inflammation-driven tumorigenesis. UMEs marked with asterisks (*) exhibit context-dependent or bidirectional effects depending on cell type, inflammatory stimulus, or disease stage.

Unlike E2 enzymes that primarily tune activation thresholds, E3 ligases control signal directionality and strength, thereby shaping distinct cellular programs. In intestinal inflammation, they coordinate stress responses, survival, and cell death across multiple signaling axes, forming a multilayered regulatory framework that links molecular events to tissue-level outcomes (**Figure 2C, Figure 4A, Table 4**). Structurally, the E3 ligases discussed encompass major classes, including RING, HECT, RBR, and non-classical types 44.

#### Hierarchical regulation of E3-mediated cell fate

As illustrated in **Figure 2C**, E3 ligases are distributed across epithelial, innate, and adaptive immune compartments, forming an integrated regulatory network that coordinates barrier repair, immune activation, and inflammatory resolution in intestinal inflammation 19, 45 (**Figure 2C, Table 4**).

The colonic epithelium is composed of multiple specialized intestinal epithelial cell populations, including colonocytes, goblet cells, enteroendocrine cells, and stem cells (**Figure 2A**). Within IECs, representative E3 ligases such as HACE1, NEDD4L, and RNF186 regulate stress-adaptive responses and barrier-associated fate programs, thereby controlling epithelial survival, regeneration, and injury susceptibility 46-49. In innate immune cells, E3 ligases, including TRIM26, TRIM31, and Pellino1/3, modulate inflammasome activation and the amplitude of inflammatory signaling, shaping the magnitude and persistence of mucosal immune responses 50-53. In adaptive immune cells, representative E3 ligases, including ITCH, RNF5, TRAF5, TRIM21, and UHRF1, cooperatively sustain mucosal immune homeostasis by regulating Th1/Th17 differentiation, limiting pro-inflammatory signaling, and stabilizing Treg function, thereby preventing colitis exacerbation and preserving epithelial barrier integrity 54-58. Together, E3 ligases form a hierarchical, cross-compartment regulatory network that links epithelial repair, innate inflammatory amplitude, and adaptive immune tolerance, thereby coordinating inflammation resolution and tissue protection in intestinal inflammation.

#### Epithelial cell fate: balancing survival and death to maintain barrier homeostasis

Within an inflammatory microenvironment, intestinal epithelial cells continuously navigate a dynamic balance between survival and death, with E3 ligases acting as central regulators of this decision-making network 59. TNF-α can trigger epithelial apoptosis; however, UHRF1, HACE1, cIAP1, and RNF5 promote pro-survival programs by catalyzing ubiquitination at key signaling hubs (e.g., RIPK1 and TRAF2), thereby strengthening NF-κB-dependent survival signaling and shifting cell fate toward anti-apoptotic pathways 46, 56, 60, 61. In contrast, a subset of E3 ligases directly modulates stress-associated death programs: Hrd1 protects the epithelium by alleviating ER stress and limiting inflammatory amplification 62, whereas RNF183 enhances stress-linked apoptotic signaling and exacerbates mucosal injury 63. Beyond canonical apoptosis control, E3 ligases further shape epithelial fate through multiple adaptive stress-response pathways. In ferroptosis regulation, NEDD4L induces epithelial ferroptosis by suppressing the SLC3A2-GPX4 axis, thereby restricting aberrant proliferation and impeding the transition from inflammation to colorectal cancer 47. In autophagy control, Parkin promotes autophagy-lysosome-dependent degradation of VDR, dampening vitamin D signaling and compromising barrier homeostasis 64. Conversely, MARCH8, RNF8, and RNF128 support epithelial stability by driving selective autophagy to eliminate inflammation-associated substrates, thereby restraining inflammatory spread 65-67. E3 ligases also exert bidirectional control over oxidative stress adaptation. RINCK and RNF31 suppress NRF2-mediated antioxidant programs, increase ROS accumulation, and aggravate epithelial injury 68, 69. In contrast, Hakai and TRIM59 enhance NRF2-dependent antioxidant and metabolic homeostatic responses to buffer oxidative stress, maintain barrier function, and limit persistent inflammation 70, 71. In parallel, MARCH3, TRIM21, and TRIM27 target inflammatory receptors and key nodes in innate immune signaling, whereas TRIM34 in goblet cells suppresses feed-forward inflammatory cascades, thereby sustaining the epithelial-immune interface 72-75. Collectively, the epithelial E3 ligase network integrates apoptosis, autophagy, ferroptosis, and oxidative stress pathways to form a central regulatory hub for epithelial fate decisions under inflammatory stress, thereby shaping barrier homeostasis and influencing disease trajectories.

#### Innate immune cell fate: inflammasome-driven pyroptosis and functional polarization

During intestinal inflammation, innate immune cells undergo both programmed pyroptosis and functional polarization, and E3 ligases serve as key determinants of these fate transitions. TRIM31 restrains inflammasome activation and pyroptosis by promoting ubiquitination and degradation of NLRP3 51. In contrast, RNF31 enhances NLRP3 inflammasome activity via ubiquitin-dependent mechanisms, amplifying inflammatory output and exacerbating tissue damage 76. In macrophage polarization, Pellino1 drives pathological polarization by augmenting STAT3-dependent inflammatory signaling 52, whereas TRIM33 promotes inflammation resolution by supporting monocyte differentiation and facilitating the M1-to-M2 transition 77. Moreover, c-Cbl, MARCH3, TRAF2, TRAF3, and UHRF1 collectively restrain excessive cytokine production by tuning signaling thresholds at inflammatory receptors and regulating the NF-κB/IRF transcriptional axis, thereby maintaining mucosal innate immune homeostasis 60, 72, 78, 79. Conversely, Pellino3, RNF40, TRAF4, TRAF6, TRIM14, and TRIM26 act as signal amplifiers that increase TLR/NOD pathway output 50, 53, 80-82, whereas cIAP1/2, RNF99, RNF138, TRIM27, and TRIM58 impose negative feedback to limit inflammatory signaling strength 83-87. In summary, these opposing modules define the magnitude of innate immune responses and thereby influence the persistence of intestinal inflammation and the risk of malignant progression. Overall, E3 ligases fine-tune innate immune fate by bidirectionally regulating inflammasome activity, polarization circuits, and receptor-centered inflammatory signaling networks.

#### Adaptive immune cell fate: lineage specification and immune tolerance

In the adaptive immune compartment, E3 ligases govern T-cell lineage specification and immune tolerance by controlling the stability of transcription factors and the intensity of upstream signaling. ITCH suppresses Th17 differentiation by ubiquitinating and promoting the degradation of RORγt, while also contributing to the Treg program 54, 55. Loss of ITCH results in excessive Th17 expansion, impaired barrier repair, and immune imbalance, thereby driving spontaneous colitis and increasing cancer risk 54, 55. UHRF1 exerts cell-type-dependent effects in intestinal inflammation. It limits Treg differentiation by maintaining DNA methylation, and its loss attenuates colitis in T cell transfer models 88. In contrast, UHRF1 restrains TNF-α expression in macrophages via promoter methylation, and its deficiency aggravates DSS-induced colitis 60. In addition, RNF5, TRAF5, and TRIM21 cooperatively restrain pathogenic Th1/Th17 effector programs and limit excessive adaptive immune activation; deficiency in these regulators exacerbates CD4⁺ T-cell-driven colitis phenotypes 56-58.

#### Tissue-level regulation of disease outcomes: from inflammatory homeostasis to a cancer-permissive state

At the tissue level, E3 ligases coordinate epithelial homeostasis with innate and adaptive immune responses, thereby critically influencing whether inflamed colonic tissue resolves back to homeostasis or progresses into chronic inflammation. This tissue-scale decision subsequently shapes the long-term risk of CAC. Several E3 ligases, including MARCH3, RNF128, RNF138, and TRIM21, inhibit the colitis-to-cancer transition by suppressing IL-6-STAT3 signaling, constraining aberrant epithelial proliferation, and/or dampening NF-κB activity 72, 73, 85, 89. In contrast, TRIM27 promotes this process 74. Clinical observations further support this directional regulation, with the former group frequently downregulated in IBD or CRC tissues 72, 73, 85, 89, whereas TRIM27 is markedly upregulated in CRC specimens, consistent with their opposing functions along the inflammation-tumor fate axis 74.

It should be noted that most of the regulatory mechanisms summarized in this section are derived from inflammation-driven experimental systems, including chemically induced models such as azoxymethane/dextran sulfate sodium (AOM/DSS) and genetically engineered models of chronic colitis 47, 90. These models primarily recapitulate key pathological features of CAC, including persistent inflammatory stress, epithelial barrier disruption, immune microenvironment remodeling, and inflammation-dependent tumor initiation and progression. In contrast, sporadic colorectal cancer is predominantly driven by oncogenic mutations and develops within a distinct etiological and microenvironmental context. Therefore, while certain ubiquitin-dependent regulatory pathways may be shared, caution should be exercised when extrapolating inflammation-centered mechanisms directly to mutation-driven sporadic CRC.

In parallel, TRIM34, HACE1, ITCH, and NEDD4L restrict the establishment of a pro-tumorigenic premalignant microenvironment by maintaining epithelial stability and/or restraining excessive immune activation 46, 47, 54, 75. Conversely, Pellino1, Pellino3, and TRIM14 promote chronic inflammatory maintenance and amplify tumor-promoting signaling cascades 52, 53, 82. Collectively, these antagonistic E3 ligases form a dynamic regulatory network at the tissue scale, orchestrating the balance between inflammatory resolution and cancer-permissive transformation.

#### Fate “integrators” across cell types and signaling layers: ITCH and RNF186

A subset of E3 ligases exerts coordinated control across multiple cell types and signaling layers, functioning as fate “integrators.” Here, ITCH and RNF186 exemplify this role. ITCH coordinately regulates adaptive immunity, epithelial repair, and fibrotic fate by promoting degradation of RORγt and HIC-5, thereby balancing Th17 differentiation, epithelial homeostasis, and fibroblast activation, ultimately constraining inflammation-associated carcinogenesis and intestinal fibrosis 54, 55, 90. RNF186 plays bidirectional roles in intestinal inflammation: it can promote ER stress-associated apoptosis via BNip1 ubiquitination 91, yet it also preserves mucosal homeostasis by supporting EPHB2-dependent autophagy, ATF6-UPR signaling, and Occludin-mediated tight-junction proteostasis 48, 49, 92; loss-of-function variants compromise these protective arms and increase colitis susceptibility 48, 49, 92.

Together, the hierarchical regulation of cell fate by E3 ubiquitin ligases provides a unifying framework linking inflammation to malignant progression. At the cellular level, these ligases dictate epithelial survival versus death, macrophage pyroptosis versus polarization, and T-cell effector versus regulatory differentiation. At the tissue level, these micro-scale fate choices are integrated into macro-scale outcomes, determining whether inflammation resolves or persists** (Figure 4A).** Dysregulated E3 activity disrupts this equilibrium, driving immunopathology and/or promoting tumorigenesis. Thus, the ubiquitin system not only controls protein turnover but, through cross-scale regulatory networks, ultimately shapes the clinical fate of colonic tissue—healing, chronic inflammation, or malignant transformation.

### Hierarchical regulation of cell fate by DUBs

DUBs counterbalance ubiquitin-dependent signaling to fine-tune inflammatory responses in the gut 93, 94. By removing ubiquitin chains from key regulators, DUBs modulate protein stability, activity, and localization, thereby shaping the behavior of epithelial and immune cells under stress. Rather than merely buffering signals, DUBs construct multilayered regulatory circuits that restore signaling thresholds, resolve inflammation, and preserve cellular equilibrium. In intestinal inflammation, they orchestrate fate decisions across epithelial, innate, and adaptive immune compartments, ultimately impacting whether tissue regains homeostasis or progresses toward chronic inflammation and malignancy. DUBs fall into four major families—USP, OTU, JAMM, and MJD, each defined by distinct catalytic architectures and chain-linkage specificities 94-96 (**Figure 2D, Figure 4B, Table 5**).

#### Hierarchical regulation of DUB-mediated cell fate

As shown in **Figure 2D and Table 5**, DUBs are distributed across intestinal epithelial, innate immune, and adaptive immune compartments, forming a cross-cell type regulatory network that coordinately balances inflammatory responses and tissue repair.

In IECs, OTULIN, USP9X, USP13, USP22, USP25, and USP47 maintain barrier homeostasis and attenuate colitis progression by suppressing NF-κB, STAT3, and inflammation-related signaling pathways 25, 97-101. In contrast, USP7 enhances oxidative stress and exacerbates inflammatory responses under specific pathological conditions 102, whereas A20 and CYLD exhibit context-dependent dual regulatory roles 103-106. In innate immune cells, JOSD2, CYLD, A20, OTUD1, and USP38 exert anti-inflammatory effects by limiting inflammatory signal amplification and reducing tissue damage 107-110. By comparison, BRCC3, OTUD5, OTUD6A, USP16, and USP25 promote immune activation through enhanced cytokine production, thereby modulating the magnitude of inflammatory responses 111-115. In adaptive immune cells, CYLD, USP7, USP8, USP21, and USP28 regulate T cell activation and the Th17/Treg differentiation balance, maintaining mucosal immune homeostasis and restraining excessive inflammation 109, 116-119. Conversely, sCYLD promotes inflammatory signaling activation in T cells and enhances pro-inflammatory responses 120. Collectively, DUBs establish a compartmentalized and reversible deubiquitination regulatory system that cooperates with E3 ligases to coordinately orchestrate inflammation resolution and tissue protection in the intestine.

#### Epithelial cell fate: balancing apoptosis and stress adaptation to maintain barrier integrity

DUBs preserve epithelial integrity by modulating apoptosis, endoplasmic reticulum (ER) stress, and oxidative responses. OTULIN and USP13 stabilize the epithelial barrier by inhibiting inflammatory signaling (e.g., NF-κB) or alleviating ER stress, thereby limiting TNF-induced or stress-driven epithelial apoptosis 25, 97. USP25 and USP13 act as anti-stress protectors by either dampening UPR signaling or sustaining STAT3 activation, contributing to epithelial cell survival and tissue repair 25, 100. In contrast, USP7 suppresses NRF2-dependent antioxidant pathways, resulting in ROS accumulation and increased vulnerability to oxidative injury 102. These enzymes collectively constitute a DUB network that calibrates epithelial stress responses and determines survival versus death outcomes under inflammatory challenge.

#### Innate immune cell fate: amplification versus suppression of inflammatory programs

DUBs determine the amplitude and persistence of inflammatory responses in innate immune cells such as macrophages. BRCC3 and OTUD6A enhance inflammasome activation by removing ubiquitin modifications from NLRP3, thereby promoting IL-1β/IL-18 secretion and pyroptosis, which amplifies inflammatory output 111, 113. USP16 reinforces pro-inflammatory activation by augmenting IKKβ-p105 signaling in macrophages, driving sustained inflammation 114. In contrast, USP38 suppresses immune overactivation by deubiquitinating H2B and stabilizing KDM5B, which limits NF-κB-dependent IL-6 and IL-23 expression 110. Taken together, these DUBs form a regulatory module that tunes the polarization state of innate immune cells and dictates inflammatory outcomes.

#### Adaptive immune cell fate: T cell differentiation, homeostasis, and effector balance

In the adaptive immune system, DUBs regulate the fate of T cell subsets such as Tregs and Th17 cells by modulating activation thresholds and maintaining transcription factor stability. OTUD5 promotes Th17 differentiation and sustains its pro-inflammatory effector functions 112. In contrast, USP8 stabilizes Tregs by enhancing the Foxo1-IL-7Rα signaling axis 117, and USP21 inhibits Th17 differentiation by constraining AhR activity 118. These DUBs collectively act within the inflammatory milieu to uphold immune tolerance and prevent dysregulated adaptive immune activation.

#### Tissue-level outcomes: inflammatory resolution versus tumor-permissive remodeling

At the tissue scale, DUBs integrate epithelial homeostasis, immune responses, and signaling adaptation to shape inflammatory outcomes, determining whether inflamed tissue resolves toward mucosal homeostasis or evolves into a tumor-permissive state. OTUD6A and USP16 promote chronic inflammatory persistence and CAC development by enhancing NLRP3 inflammasome activation or IKKβ-NF-κB signaling 113, 114. USP25 compromises mucosal antimicrobial defense and epithelial secretory lineage integrity by modulating secretory cell development and the Wnt pathway, thereby facilitating tumorigenesis 115. In contrast, JOSD2 attenuates tissue inflammatory burden by suppressing macrophage inflammatory pathways 108; CYLD limits NF-κB and JNK activation 106, USP9X supports epithelial stem/progenitor cell differentiation 98, and USP22 mitigates chronic epithelial injury through transcriptional repression 99. Collectively, these DUBs define opposing tissue-level outcomes, ranging from stable mucosal homeostasis to inflammation-driven carcinogenic remodeling.

Notably, A20 and CYLD exemplify the context-dependent dual roles of DUBs across distinct cellular environments. Deletion of A20 results in uncontrolled NF-κB activation and systemic inflammation 107, 121. In contrast, in IECs, A20 exhibits a dose-dependent bifunctional role in TNF signaling 103, 104. Loss of A20 sensitizes epithelial cells to TNF-induced apoptosis by impairing NF-κB-dependent survival programs 104, whereas excessive A20 expression paradoxically promotes RIPK1-dependent ripoptosome assembly and caspase-8 activation 103. Mechanistically, both insufficient and excessive A20 activity destabilize TNFR1 signaling homeostasis by altering the balance between membrane-associated complex I and cytosolic death-inducing complex II formation. This threshold-dependent regulatory behavior positions A20 as a rheostat rather than a unidirectional suppressor of inflammation in epithelial compartments. CYLD exhibits similar complexity: in IECs, it promotes necroptosis and epithelial barrier disruption 106, while in immune cells, it exerts anti-inflammatory effects by restricting TRAF-NEMO signaling and NLRP6 inflammasome activation 105, 109. The short splice variant sCYLD further exacerbates inflammation by inhibiting TGF-β signaling, destabilizing Treg cells, and enhancing Th1/Th17 responses 120.

Collectively, DUBs fine-tune ubiquitination landscapes to orchestrate hierarchical fate decisions within the inflammatory microenvironment. By controlling cell survival, immune balance, and tissue pathology, DUBs serve as critical nodes linking molecular signaling dynamics to disease outcomes, including resolution, chronic inflammation, or tumorigenesis.

## From cell fate modulation to pathway control: multi-layered roles of UMEs in inflammatory signaling networks

**Figure 5:**
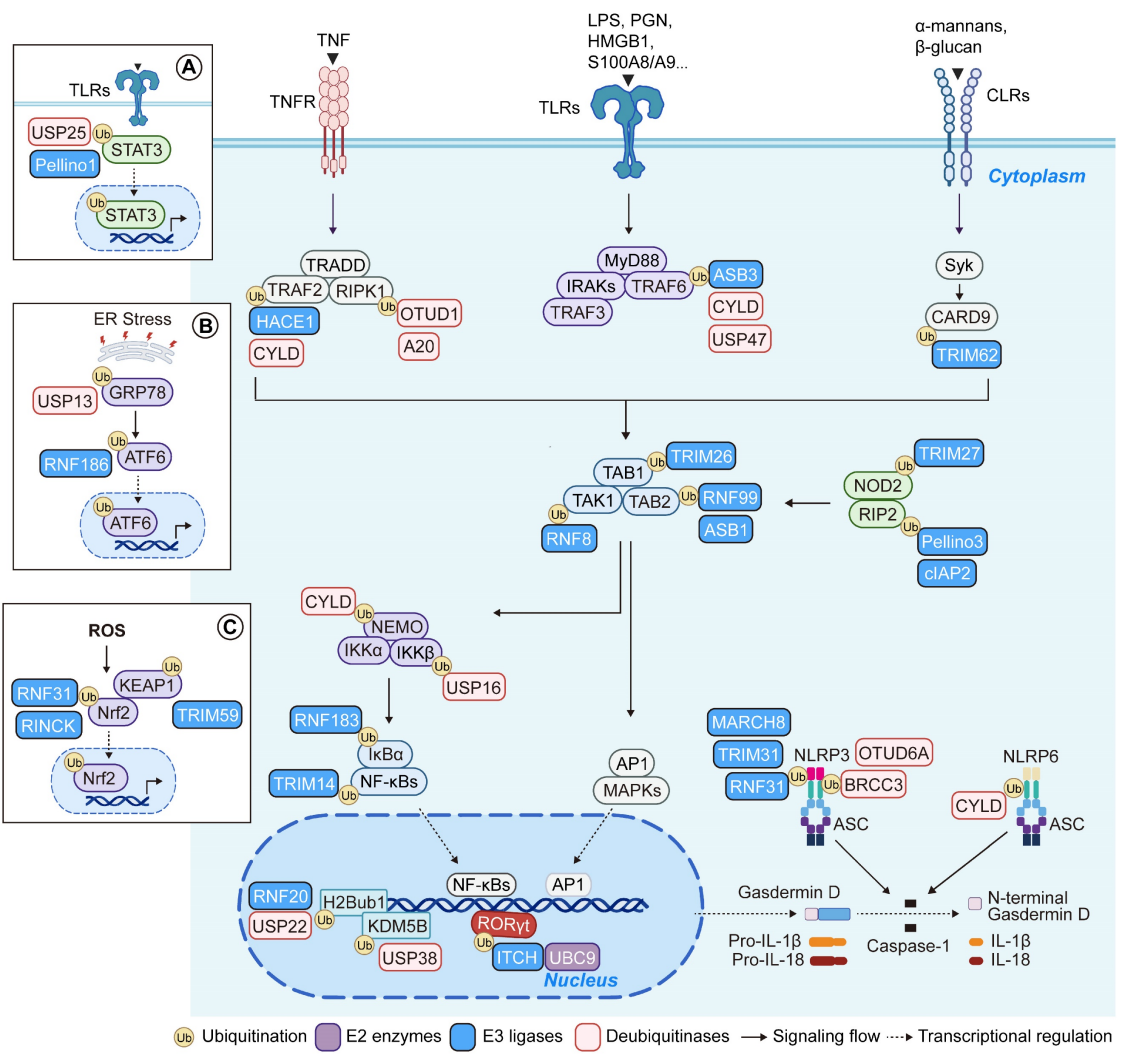
** Multi-layered regulation of inflammatory signaling networks by UMEs in intestinal inflammation.** Schematic overview illustrating how ubiquitin-modifying enzymes (UMEs), including E2 conjugating enzymes, E3 ligases, and DUBs, coordinate the regulation of key inflammatory signaling pathways in intestinal epithelial and immune cells. UMEs modulate receptor-proximal signalosome assembly (TLRs, TNFR, CLRs, and NOD2), downstream kinase cascades (TAK1-TAB, MAPKs, and IKK complexes), transcriptional programs (NF-κB, AP-1, STAT3), stress-responsive pathways (ER stress and oxidative stress signaling), and inflammasome activation (NLRP3/NLRP6). These coordinated regulatory layers collectively shape cell fate outcomes, including inflammatory activation, stress adaptation, programmed cell death, and barrier homeostasis. Blue boxes indicate E3 ligases, red boxes indicate DUBs, and green boxes represent E2 enzymes. Solid arrows denote signaling flow, whereas dashed arrows indicate transcriptional regulation. Created in BioRender. Qian, C. (2026) https://BioRender.com/a9pj9u4.

Following recognition of the layered role of UMEs in shaping cell-fate decisions, accumulating evidence reveals their equally critical function in precisely modulating key molecular nodes within well-defined inflammatory signaling pathways **(Figure 5, Tables 3-5)**. In intestinal inflammation, UMEs dynamically regulate signal transduction complexes, modulate adaptor protein activity, and control the stability of transcription factors across core axes such as TLRs, TNFRs, NOD-like receptors, and STAT signaling, thereby shaping the amplitude, duration, and outcomes of immune responses.

Pattern recognition receptors (PRRs), including TLRs, CLRs, and NOD2, detect microbial and damage-associated molecular patterns and rapidly recruit adaptor proteins such as TRADD, MyD88, IRAKs, and RIP2 to form signaling complexes. These complexes are finely regulated by various E3 ligases (such as Pellino3, ASB3, TRIM62, and cIAP2) and DUBs (including CYLD, USP47, A20, and OTUD1), which together orchestrate signal amplification or termination to balance pro-inflammatory responses with negative feedback control 83, 101, 103, 109, 122-126. Signals from diverse receptors converge on the TAK1-TAB complex, a key signaling hub whose assembly and activation are modulated by E3 ligases including TRIM26, RNF8, RNF99, and ASB1, thereby allowing graded control over NF-κB and MAPK activation and transcriptional responses 50, 66, 84, 127.

TLR signaling also intersects with STAT3 to influence epithelial-immune crosstalk. Pellino1 promotes STAT3 ubiquitination and nuclear translocation, while USP25 stabilizes STAT3 via deubiquitination, linking inflammatory signaling with epithelial survival 52, 100. Beyond canonical immune pathways, stress-related signaling is also tightly regulated by the ubiquitin system. Under endoplasmic reticulum stress, RNF186 and USP13 modulate the ubiquitination of GRP78 and ATF6, respectively, adjusting the unfolded protein response and gene expression 25, 92. In the context of reactive oxygen species (ROS) accumulation, RNF31 and RINCK suppress NRF2 activation, thereby promoting oxidative damage and inflammation 68, 69. Conversely, TRIM59 enhances KEAP1 degradation, facilitates NRF2 nuclear translocation, and upregulates antioxidant gene expression, mitigating epithelial injury and restraining colitis progression 71. At the transcriptional level, RNF20, USP22, and USP38 converge on epigenetic regulation to repress inflammatory gene programs 99, 110, 128. UMEs also govern inflammasome activity: E3 ligases such as RNF31, TRIM31, and MARCH8, along with DUBs like BRCC3, OTUD6A, and CYLD, modulate NLRP3 and NLRP6 ubiquitination, thereby influencing caspase-1 activation, Gasdermin D cleavage, and IL-1β/IL-18 maturation, which shape pyroptotic responses and inflammatory amplification 51, 65, 76, 105, 111, 113. In adaptive immunity, ITCH suppresses Th17 differentiation by ubiquitinating and degrading RORγt; its deficiency leads to exaggerated NF-κB activity and spontaneous colitis. In parallel, hypoxia represses UBC9 transcription, reducing RORγt SUMOylation and enhancing IL-17 expression, thereby reinforcing Th17-driven pathogenicity 41, 55. Altogether, UMEs operate across multiple layers of the intestinal inflammatory network, ranging from signalosome assembly to transcriptional control, to define the trajectory of immune activation, tissue injury, and homeostatic resolution, making them pivotal regulators of inflammation and disease fate.

## Emerging hotspots and future directions of UME regulation in IBD

**Figure 6:**
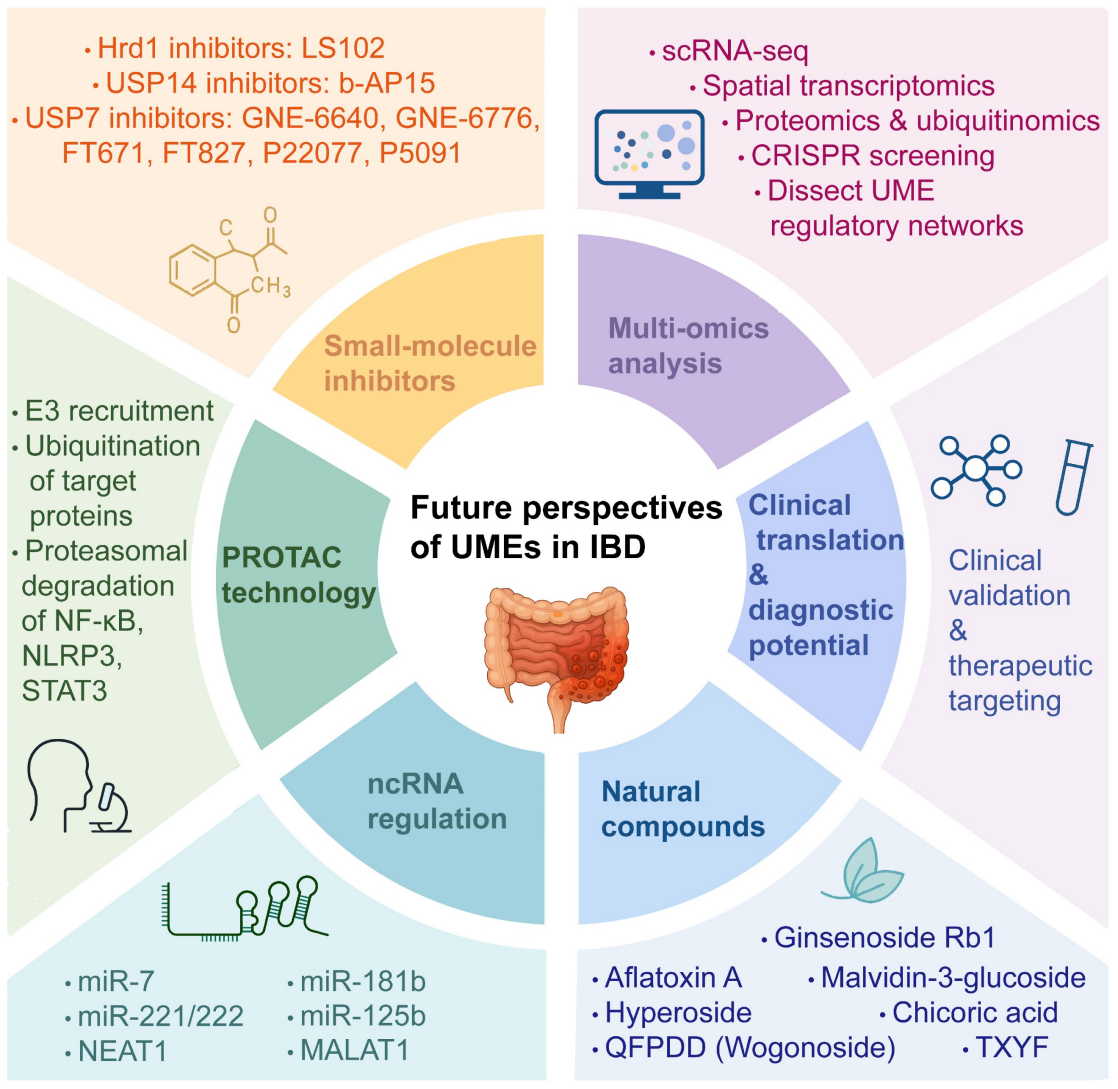
** Future perspectives of UMEs in IBD.** Schematic summary of emerging research directions and therapeutic strategies targeting ubiquitin-modifying enzymes (UMEs) in inflammatory bowel disease, including small-molecule inhibitors, PROTAC technology, ncRNA-mediated regulation, natural compounds, multi-omics approaches, and clinical translational potential. Created in BioRender. Qian, C. (2026) https://BioRender.com/7t7alxc.

Building on the expanding mechanistic understanding of UME function in intestinal inflammation, efforts are now increasingly directed toward developing therapeutic and technological strategies to modulate UME activity in IBD (**Figure 6**).

### Development of small-molecule modulators targeting UMEs

With the increasing recognition of UMEs as pivotal regulators in chronic inflammatory diseases, small-molecule inhibitors targeting DUBs and E3 ligases have become a major research focus. Among them, USP7 and USP14, which regulate NF-κB signaling, inflammatory gene expression, and epithelial barrier homeostasis, have emerged as promising therapeutic targets in IBD.

#### Structural and functional diversity of USP7 small-molecule inhibitors

The therapeutic targeting of USP7 has evolved from early non-specific covalent inhibitors to highly selective allosteric modulators, driven by a deeper understanding of its complex structural dynamics 129, 130. USP7 transitions between an inactive (apo) state and an active (ubiquitin-bound) state through significant rearrangements of its catalytic triad (Cys223, His464, and Asp481) 129.

Recent studies have identified small molecules that exploit USP7 conformational dynamics to achieve selective inhibition. GNE-6640 and GNE-6776 act as non-covalent inhibitors that bind near the catalytic cleft, induce rearrangement of the switching loop, and prevent ubiquitin engagement, thereby suppressing USP7 activity in cancer models 130. In contrast, FT671 and FT827 selectively stabilize the apo conformation of USP7, block catalytic triad realignment, and promote MDM2 degradation, leading to restoration of p53 tumor-suppressive signaling 129. Beyond oncology, USP7 has also emerged as a critical regulator of inflammatory signaling and redox homeostasis. P22077, an early-generation dual inhibitor of USP7 and USP47, exerts anti-inflammatory effects by targeting multiple signaling pathways. Mechanistically, P22077 promotes K48-linked ubiquitination and degradation of TRAF6, thereby attenuating LPS-induced TLR4/NF-κB activation 131. In addition, USP7 inhibition by P22077 suppresses NLRP3 inflammasome activation by preventing ASC oligomerization and speck formation in a transcription-independent manner 132.

In the specific context of IBD, USP7 plays a deleterious role by modulating the intestinal redox state. Recent evidence highlights that USP7 stabilizes AMBRA1; the accumulated AMBRA1 then antagonizes DUB3-mediated deubiquitination of NRF2, leading to NRF2 degradation and increased oxidative stress in IECs. Consequently, inhibition of USP7 by P5091 or P22077 restores the NRF2-driven antioxidant response and alleviates mucosal inflammation in experimental colitis models 102, 133. These findings suggest that USP7 inhibitors do not merely suppress inflammation but also act as multifaceted regulators of epithelial barrier integrity and oxidative balance, making them promising candidates for IBD therapy.

#### Small-molecule inhibitors targeting proteasome-associated USP14

USP14 is a major deubiquitinating enzyme associated with the 19S regulatory particle of the 26S proteasome and plays an essential role in fine-tuning proteasomal protein degradation. Unlike most DUBs, inhibition of USP14 directly interferes with proteasomal substrate processing, making it a potential therapeutic target in diseases characterized by proteostasis imbalance and inflammatory dysregulation.

b-AP15 is a potent small-molecule inhibitor targeting both USP14 and UCHL5. Its inhibition leads to rapid accumulation of high-molecular-weight polyubiquitinated proteins, thereby inducing pronounced proteotoxic stress 134. In contrast to classical proteasome inhibitors, b-AP15-induced apoptosis is closely associated with enhanced oxidative stress and ROS generation, accompanied by mitochondrial membrane potential loss and structural damage, ultimately driving programmed cell death 135. Beyond its pro-apoptotic effects in cancer models, b-AP15 also exhibits anti-inflammatory activity. USP14 inhibition attenuates LPS-induced inflammatory responses by suppressing ERK1/2 and JNK signaling and limiting NF-κB nuclear translocation 136. By simultaneously modulating proteostasis and inflammatory signaling networks, targeting USP14 represents a multifaceted therapeutic strategy for inflammatory disorders.

#### Other small-molecule inhibitors targeting UMEs

Beyond the USP family, pharmacological modulation of other UMEs, particularly E3 ligases involved in ER stress and epithelial proteostasis, has also been explored. Among these, Hrd1 has attracted attention due to its critical role in regulating unfolded protein response signaling and maintaining epithelial stress tolerance. Experimental inhibition of Hrd1 using LS102 markedly exacerbates colitis severity in murine models, highlighting its protective function in limiting ER stress-driven epithelial injury and inflammatory amplification 62.

Collectively, these studies demonstrate that small-molecule modulation of UMEs can influence intestinal inflammation through multiple mechanisms, including inflammatory signal transduction, redox homeostasis, proteostasis regulation, and epithelial stress adaptation. While USP7 and USP14 inhibitors primarily act by reshaping inflammatory and proteotoxic signaling networks, targeting stress-responsive E3 ligases, such as Hrd1, underscores the importance of preserving epithelial homeostatic capacity. Together, these findings underscore both the therapeutic potential and the inherent complexity of UME-targeted strategies in IBD. Rational drug design will therefore require careful consideration of target selectivity, cell type specificity, and tissue-context dependence to balance anti-inflammatory efficacy with the maintenance of intestinal barrier integrity.

### Natural compounds in the regulation of UMEs

Natural products are gaining attention as complementary regulators of UMEs. These bioactive compounds can influence UME expression or activity, thereby modulating intestinal inflammation, oxidative stress, and mitophagy.

For instance, in humanized colitis models, DSS or TNBS challenge significantly downregulated Hrd1 expression while upregulating ER stress markers such as GRP78, PERK, CHOP, and caspase-12. Treatment with ginsenoside Rb1 reversed these changes, suggesting a role in alleviating ER stress by modulating Hrd1 62. Similarly, aflatoxin A has been reported to promote the progression of chronic colitis and colorectal cancer by targeting the RINCK signaling pathway 137, underscoring the involvement of UMEs in IBD-associated tumorigenesis. Moreover, the traditional Chinese formula Tongxie Yaofang (TXYF) suppressed tumor formation in a CAC mouse model. TXYF not only inhibited the proliferation of LPS-stimulated epithelial and colorectal cancer cells but also blocked epithelial-mesenchymal transition (EMT) by activating PINK1/Parkin-dependent mitophagy, thereby exerting both anti-inflammatory and antitumor effects 138.

Among single compounds, Hyperoside was found to suppress MKRN1 expression, thereby enhancing ubiquitin-mediated degradation of its substrate, PPARγ, ultimately ameliorating DSS-induced colitis in mice 139. Licorice extract has been shown to activate the Nrf2/PINK1 pathway, promoting mitophagy and reducing inflammatory cytokine production and oxidative stress in UC models 140. Another study demonstrated that the blueberry anthocyanin malvidin-3-glucoside (MG) upregulated HACE1, restored gut microbial homeostasis, and increased the abundance of beneficial bacteria such as Bifidobacteria, conferring protection in colitis models 141. Likewise, Qingfei Paidu Decoction (QFPDD) and its bioactive component wogonoside suppressed USP14 expression while promoting ATF2 degradation, resulting in reduced IL-6 and TNF-α production, enhanced IL-10 expression, and alleviation of intestinal inflammation 142. Furthermore, Chicoric acid was shown to ameliorate DSS-induced colitis by targeting the USP9X/IGF2BP2 axis 143.

### Regulation by long non-coding RNAs or microRNAs

In addition to classical regulation at the protein level, increasing evidence indicates that UMEs are also subject to fine-tuned control by non-coding RNA (ncRNA) networks 144. Specific microRNAs (miRNAs) and long non-coding RNAs (lncRNAs) can modulate the transcription, mRNA stability, or translational efficiency of UME-encoding genes, thereby indirectly influencing downstream signaling functions. Such regulatory layers play important roles in the onset and maintenance of intestinal inflammation 145, 146.

For instance, miR-7 suppresses RNF183 expression and alleviates DSS-induced colitis 147. Conversely, miR-221/222 enhances NF-κB and STAT3 signaling activity through dual mechanisms: directly stabilizing RelA mRNA and indirectly repressing PDLIM2 (an E3 ligase that promotes the degradation of RelA and STAT3), thereby amplifying proinflammatory signals. Targeting miR-221/222 efficiently disrupts this pathogenic loop and inhibits colorectal cancer cell proliferation 148. Furthermore, several miRNAs (e.g., miR-125b, miR-181b) and lncRNAs (e.g., NEAT1, MALAT1) have been experimentally validated to regulate key UMEs (such as A20, CYLD, and TRAF6), thereby modulating NF-κB signaling, cell survival, and immune homeostasis 149-153.

Although research on ncRNA-mediated regulation of UMEs in the context of IBD is still in its infancy, these findings highlight ncRNAs as upstream regulators of the UME network. Together, these findings expand our mechanistic understanding of UME regulation. Future studies integrating multi-cellular transcriptome profiling and ncRNA-UME interaction networks may help construct a multi-layered regulatory model, ultimately providing a more comprehensive explanation of UME functions in IBD.

### Emerging PROTAC technology for targeting UMEs

In recent years, proteolysis-targeting chimera (PROTAC) technology has attracted considerable attention as a promising strategy for small-molecule drug development due to its ability to induce selective protein degradation 154. Unlike conventional inhibitors that merely block enzymatic activity, PROTAC recruits an E3 ligase to ubiquitinate the target protein, thereby directing it toward proteasomal degradation. This provides a novel avenue for targeting “undruggable” proteins 155. Although direct studies on UMEs in the context of IBD are still lacking, the successful application of PROTAC in oncology, autoimmunity, and other disease areas provides a strong theoretical basis for its potential application in IBD.

Of particular note, many UME-regulated proteins are highly stable and thus challenging to inhibit directly (e.g., certain DUBs or catalytically inactive E3 ligases), making them attractive PROTAC targets 156. Future design strategies may consider developing PROTAC molecules against key IBD-related inflammatory mediators (such as NF-κB subunits, NLRP3, or STAT3). By harnessing E3 ligase-mediated selective degradation, such approaches could achieve precise regulation of inflammatory pathways. Furthermore, combining PROTAC platforms with tissue-specific delivery systems or biomaterial-based organ targeting strategies may further enhance their translational potential and therapeutic efficacy in IBD.

In summary, although PROTAC-based approaches are still in the early stages of research on intestinal inflammation, their mechanistic foundation in ubiquitin signaling highlights the promise of PROTAC as a powerful tool for modulating UMEs. This strategy may open new avenues for therapeutic intervention in IBD pathogenesis.

### Multi-omics approaches for dissecting UME regulatory networks

The rapid development of multi-omics technologies has provided new perspectives for elucidating the regulatory roles of UMEs in IBD. Single-cell RNA sequencing (scRNA-seq) and spatial transcriptomics have revealed cell-type- and tissue-specific expression patterns of UMEs, enabling precise localization of their activity in diseased tissues 157-159. Integration of proteomic, ubiquitinomic, and transcriptomic data will facilitate the construction of comprehensive molecular regulatory networks of UMEs in intestinal immune inflammation, barrier dysfunction, and cell death. In addition, high-throughput CRISPR/Cas9 functional screens offer the opportunity to systematically identify essential UMEs and define their cell type- or pathway-specific roles. Together, these approaches will accelerate mechanistic dissection and uncover new therapeutic targets in IBD 160-162.

### Clinical translation and diagnostic potential

**Figure 7:**
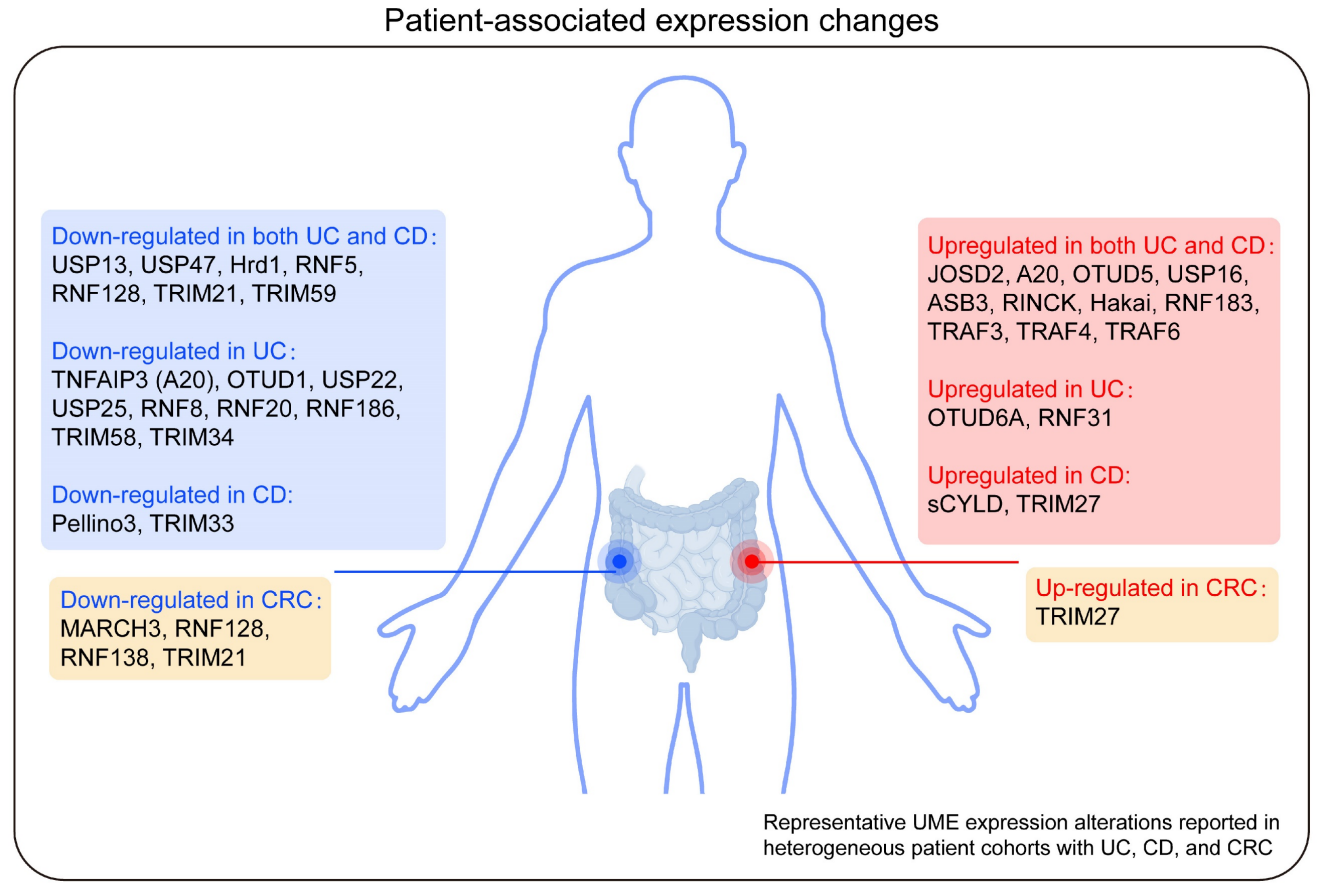
** Representative UME expression alterations reported in heterogeneous patient cohorts with UC, CD, and CRC.** Schematic summary of ubiquitin-modifying enzymes (UMEs) that are differentially expressed in ulcerative colitis (UC), Crohn's disease (CD), and colorectal cancer (CRC) based on reported transcriptomic and tissue-level expression analyses. Blue boxes indicate UMEs that are downregulated, whereas red boxes indicate UMEs that are upregulated in the indicated disease conditions. Enzymes altered in both UC and CD are shown separately from those selectively dysregulated in individual disease subtypes. The CRC-associated panel summarizes UMEs with consistent expression changes during colorectal tumorigenesis. Created in BioRender. Qian, C. (2026) https://BioRender.com/o0txak6.

Current evidence supporting the involvement of UMEs in IBD is primarily derived from heterogeneous human datasets, including bulk transcriptomic profiling, protein-level measurements, peripheral blood biomarker analyses, and genome-wide association studies (GWAS) 163, 164. As summarized in the “Alteration in patients” category, many UMEs exhibit disease-associated expression changes in tissues from patients with UC, CD, or CRC **(Figure 7, Tables 3-5)**. For example, UBC9, TRIM59, and USP13 are downregulated in patients with UC and CD and exhibit significant correlations with disease activity, indicating that alterations in UME expression may serve as indicators of disease progression or predictors of therapeutic response 25, 42, 71. However, it should be emphasized that most patient-derived data reflect correlative associations rather than direct causal relationships. In contrast, only a subset of UMEs has been functionally validated in disease-relevant experimental systems, particularly inflammation-driven models such as AOM/DSS-induced CAC or genetically engineered colitis models. Therefore, caution is warranted when inferring mechanistic roles or therapeutic target potential based solely on observational human data. Future studies should prioritize the systematic validation of UMEs as biomarkers and companion diagnostics, alongside mechanistic investigations to establish their direct causal contributions to epithelial dysfunction, immune dysregulation, and inflammatory disease progression. Collectively, integrating multi-layer human datasets with functional validation in inflammation-driven models will be essential for refining the translational relevance of UMEs and prioritizing high-confidence targets for precision intervention in IBD and related inflammatory pathologies.

## Conclusion, limitations, and future perspectives

By functioning as central regulators of protein homeostasis, UMEs play fundamental roles in controlling cell fate decisions during intestinal inflammation. By coordinating programmed cell death, stress adaptation, and survival signaling across epithelial and immune compartments, UMEs critically shape tissue homeostasis and inflammatory outcomes in IBD. This review has synthesized the current understanding of the expression dynamics and functional properties of E2 enzymes, E3 ligases, and DUBs, highlighting how ubiquitin-dependent mechanisms integrate cell-type-specific signaling networks under inflammatory stress.

Despite substantial progress, major conceptual and technical challenges remain in understanding UME-mediated regulation of intestinal inflammation. Key unresolved questions include the substrate specificity of individual UMEs, the functional selectivity of distinct ubiquitin chain architectures, tissue- and cell-type-dependent heterogeneity, and crosstalk with transcriptional and metabolic programs. In addition, the roles of UMEs in non-epithelial stromal populations, such as fibroblasts and endothelial cells, remain largely unexplored, particularly in the context of inflammation-associated tissue remodeling and disease complications. Limitations of current experimental models should also be considered. DSS- and TNBS-induced colitis models capture specific inflammatory features but do not fully recapitulate the chronic relapsing course, genetic diversity, and clinical heterogeneity of human IBD. DSS models primarily reflect epithelial injury and innate immune activation, whereas TNBS models emphasize hapten-driven, T cell-mediated inflammation. Likewise, AOM/DSS models preferentially represent colitis-associated tumorigenesis rather than sporadic colorectal cancer driven by oncogenic mutations. These limitations underscore the need for cautious translational interpretation and complementary validation across experimental systems.

Importantly, accumulating evidence indicates that UME function is highly stage-dependent along the trajectory of intestinal inflammation. During disease initiation, UMEs mainly shape epithelial barrier integrity, early innate immune activation, and acute epithelial cell death programs. In chronic inflammation, long-term regulation of inflammatory signaling, stress adaptation, and immune cell polarization contributes to disease persistence and tissue remodeling. In contrast, during CAC progression, dysregulation of epithelial survival pathways, genomic stability, and microenvironmental signaling becomes the dominant determinant of malignant progression. As summarized in **Figure 4**, many E3 ligases and DUBs exhibit context-dependent and bidirectional roles across epithelial, innate immune, adaptive immune, and tissue-level outcome layers. This stage-resolved perspective provides a conceptual framework for reconciling divergent experimental observations and highlights the importance of disease-stage stratification when interpreting UME function. From a translational standpoint, these findings suggest that therapeutic strategies targeting UMEs may require stage-stratified and context-adapted modulation rather than uniform inhibition or activation. For example, transient suppression of pro-inflammatory UMEs may be beneficial during acute inflammation, whereas preservation of epithelial-protective and stress-adaptive UMEs may be critical during chronic disease maintenance and CAC prevention.

Looking forward, integrating multi-omics profiling, spatial transcriptomics, and single-cell technologies will enable systematic mapping of ubiquitin-regulated cell-fate networks within complex inflammatory microenvironments. Combined with emerging chemical biology approaches, including small-molecule modulators and PROTAC-based strategies, these advances will facilitate mechanistic insight into cell death regulation and stress-responsive adaptation. Together, such efforts are anticipated to provide a robust conceptual framework for prioritizing high-confidence therapeutic targets and advancing precision, mechanism-driven interventions for inflammatory diseases.

## Figures and Tables

**Table 1:** Operational definition of cell-fate programs in intestinal inflammation.

Cell fate layer	Operational definition	Representative processes	Pathological relevance	Reference
Epithelial fate	Determines epithelial survival, elimination, and regenerative capacity under inflammatory stress	Apoptosis, necroptosis, pyroptosis, ferroptosis, intestinal stem cell renewal and differentiation, barrier restitution	Barrier disruption, impaired mucosal repair, accumulation of genetically damaged epithelial cells	9, 10
Innate immune fate	Defines functional states and inflammatory thresholds of innate immune cells	Macrophage polarization (M1/M2), inflammasome activation, inflammatory tolerance programs	Amplification or resolution of chronic inflammation, shaping tumor-promoting inflammatory microenvironment	11-13
Adaptive immune fate	Controls lineage commitment and effector status of adaptive immune cells	Th1/Th17/Treg differentiation, immune exhaustion versus effector activation	Immune imbalance, impaired immune surveillance, sustained pro-tumorigenic inflammation	14, 15

**Table 2:** Tissue-level inflammatory and carcinogenic outcomes emerging from cumulative cell-fate programs.

Tissue-level outcome state	Integrated cellular drivers	Representative tissue features	Disease relevance	Reference
Inflammatory resolution and mucosal restoration	Balanced epithelial regeneration + restrained innate immune activation + regulatory adaptive immunity	Barrier repair, reduced inflammatory infiltrates, restoration of tissue architecture	Disease remission, reduced relapse risk	16
Chronic inflammatory remodeling	Persistent epithelial injury + pro-inflammatory innate polarization + immune imbalance	Sustained inflammation, fibrosis, crypt architectural distortion, dysplasia-prone microenvironment	Disease progression, increased CAC susceptibility	17
Tumor-permissive tissue state (CAC-prone)	Aberrant epithelial survival + genomic instability + pro-tumor immune niche formation	Dysplasia, epithelial transformation, stromal remodeling, pro-angiogenic signaling	CAC initiation and malignant progression	17

**Table 3:** Roles of E2 ubiquitin-conjugating enzymes in intestinal inflammation and tumorigenesis.

Gene	Pathogenesis of IBD/CAC	Effect	Cell fate	Mechanism/major finding	Target protein classification	Cell type	Alteration in patients	Transgenic mice	Disease model	Disease Phenotype	Reference
UBE2W	Imbalance of intestinal immunity	Anti-inflammatory	Innate immune fate (inflammatory activation threshold); Epithelial fate (barrier injury susceptibility)	UBE2W limits NF-κB activation by reducing IκB and p65 phosphorylation and preventing p65 nuclear translocation, thereby lowering pro-inflammatory cytokine expression	-	-	-	*AAV2/9-Ube2w OE*	DSS	Attenuated colitis	40
UBE2L3/ UBCH7	Genetic susceptibility	-	-	-	-	-	-	-	-	-	165
UBE2A, UBE2D2, UBE2L6	Immune tolerance and inflammatory activation	-	Innate immune fate (macrophage activation versus tolerogenic state)	-	-	-	-	-	-	-	39
UBC9 (SUMO E2)	Imbalance of intestinal immunity	Anti-inflammatory; Th17 pathogenicity-restraining	Adaptive immune fate (Th17 effector programming)	Hypoxia-induced HIF-1α binds to the Ubc9 promoter CpG island and promotes DNA hypermethylation, leading to transcriptional repression of Ubc9, reduced SUMOylation of RORγt, enhanced IL-17 transcriptional activity, and reinforcement of pathogenic Th17 effector responses via hypoxia-driven epigenetic reprogramming.	RORγt (SUMOylation substrate)	CD4⁺ Th17 cells; colonic lamina propria lymphocytes	Down-regulated in UC patients	-	-	-	41
UBC9 (SUMO E2)	Defect of intestinal barrier, imbalance of intestinal immunity	Anti-inflammatory; epithelial barrier-protective	Epithelial fate (survival and barrier maintenance)	Ubc9 downregulation reduces Akt1 SUMOylation and stability, leading to enhanced NF-κB-dependent inflammatory gene expression and impaired wound-healing responses through SUMO-mediated regulation of cellular stress tolerance.	Akt1 (SUMOylation substrate)	IECs, HCT-8	Down-regulated in UC and CD biopsies	Not genetic KO; Ubc9^HyperLow^	DSS	Susceptibility to colitis	42
UBC9 (SUMO E2)	Imbalance of intestinal immunity	Pro-inflammatory	Innate immune fate (DC activation state); Adaptive immune fate (CD4⁺ T cell priming and polarization)	Ubc9-dependent SUMOylation of RBPJ stabilizes RBPJ by blocking proteasomal degradation, leading to enhanced Ciita-driven MHC class II transcription and increased antigen presentation capacity of DCs, which promotes CD4⁺ T cell activation and Th1/Th17 polarization	RBPJ (SUMOylation substrate)	DCs (BMDCs, CD11c⁺ DCs)	-	Itgax-Cre; Ubc9^f/f^ (DC-specific KO)	DSS	Attenuated colitis	43

**Table 4:** Roles of E3 ubiquitin ligases in intestinal inflammation and tumorigenesis.

E3 Type	Gene	Pathogenesis of IBD/CAC	Effect	Cell fate	Mechanism/major finding	Target protein classification	Cell type	Alteration in patients	Transgenic mice	Disease model	Disease Phenotype	Reference
HECT	HACE1	Imbalance of intestinal immunity	Anti-inflammatory; CAC initiation-suppressive	Epithelial fate (apoptosis, necroptosis); Innate immune fate (NF-κB)	HACE1 ubiquitinates TRAF2 to modulate TNFR1 complex signaling, thereby limiting TNFα-induced systemic inflammation	TRAF2 (ubiquitination substrate, K63-linked)	IECs	-	*Hace1^-/-^*	DSS; AOM/DSS	Susceptibility to colitis and CAC	46
HECT	HUWE1	-	-	Epithelial fate (goblet cell differentiation)	HUWE1 negatively regulates goblet cell generation by promoting ATOH1 degradation	-	Goblet cells	-	-	-	-	166
HECT	ITCH	Imbalance of intestinal immunity	Anti-inflammatory	Innate immune fate; Adaptive immune fate (Th17 effector programming)	ITCH deficiency leads to dysregulated gut microbiota and exaggerated T cell activation, resulting in spontaneous colitis	RORγt (ubiquitination substrate)	CD4⁺ T cells; intestinal immune cells	-	*Itch^-/-^*	DSS	Severe colitis	55
HECT	ITCH	Defect of intestinal barrier; imbalance of intestinal immunity	Anti-inflammatory; CAC initiation-suppressive	Adaptive immune fate (Th17 differentiation); Epithelial fate (barrier restitution)	ITCH ubiquitinates and degrades RORγt, suppressing Th17 differentiation and IL-17-mediated intestinal inflammation and tumorigenesis. ITCH deficiency leads to exaggerated Th17 responses and enhanced NF-κB signaling in the gut	RORγt (ubiquitination substrate, K48-linked)	CD4⁺ T cells (Th17 cells)	-	*Itch^-/-^*	DSS; AOM/DSS	Susceptibility to colitis and CAC	54
HECT	ITCH	Imbalance of intestinal immunity	Anti-fibrotic	Innate immune fate; Adaptive immune fate (Th17 differentiation)	ITCH ubiquitinates and degrades HIC-5, suppressing IL-17-driven fibroblast activation and extracellular matrix production. ITCH deficiency results in increased HIC-5 stability and exacerbated intestinal fibrosis	HIC-5 (ubiquitination substrate, K63-linked)	Intestinal fibroblasts	-	*Itch^-/-^*	DSS-induced chronic colitis/fibrosis model	Severe intestinal fibrosis	90
HECT	NEDD4L	Defect of intestinal barrier; imbalance of intestinal immunity	Anti-inflammatory; barrier-protective; CAC initiation-suppressive	Epithelial fate (ferroptosis, epithelial death, barrier restitution)	NEDD4L promotes ferroptosis in intestinal epithelial cells by maintaining the SLC3A2-GPX4 axis suppression, thereby limiting IEC proliferation, resolving inflammation, and inhibiting colorectal tumorigenesis	SLC3A2 (ubiquitination substrate, K63-linked)	IECs; intestinal organoids	Down-regulated in intestinal mucosa	*Nedd4l^+/-^*;* Nedd4l^fl/fl^ VillinCre*	DSS; TNBS; AOM/DSS	Severe colitis; prone to CAC	47
RBR	Parkin	Imbalance of intestinal immunity	Pro-inflammatory; barrier-disruptive	Epithelial fate (barrier maintenance impairment)	Parkin promotes autophagy-lysosome-mediated degradation of VDR, leading to reduced VDR signaling, impaired epithelial barrier integrity	VDR (ubiquitination substrate)	IECs	-	*Parkin^-/-^*	DSS	Attenuated colitis	64
RBR	Parkin	Imbalance of intestinal immunity	CAC initiation-suppressive	Innate immune fate	Parkin mediates ubiquitination and degradation of ITF2; NF-κB p65 competes with Parkin to stabilize ITF2, thereby suppressing NF-κB target genes and inhibiting CAC progression	ITF2 (ubiquitination substrate, K48-linked)	IECs	-	-	-	-	167
RING-like (non-classical)	ASB1	Imbalance of intestinal immunity	Pro-inflammatory	Innate immune fate	ASB1 binds and stabilizes TAB2, thereby enhancing TAK1-dependent activation of NF-κB and MAPK signaling and promoting pro-inflammatory cytokine production	TAB2 (signaling adaptor/binding partner)	BMDMs; BMDCs	-	*Asb1^-/-^*	DSS	Attenuated colitis	127
RING-like (non-classical)	ASB3	Disturbance of gut microbiota; imbalance of intestinal immunity	Pro-inflammatory	Epithelial fate; Innate/adaptive immune fate	ASB3 promotes the ubiquitination and degradation of TRAF6 in intestinal epithelial cells, leading to aberrant NF-κB activation and intestinal microbiota imbalance	TRAF6 (ubiquitination substrate, K48-linked)	IECs	Up-regulated in IBD patients	*ASB3^-/-^*;* ASB3^OE (IEC)^*	DSS	Attenuated colitis (*ASB3^-/-^*); aggravated colitis (ASB3*^OE (IEC)^)*	123
RING-like (non-classical)	c-Cbl	Imbalance of intestinal immunity; impaired tolerogenic DC function	Anti-inflammatory (restricts DSS colitis)	Innate/adaptive immune fate	c-Cbl mediates ubiquitination and degradation of RelB downstream of Dectin-2/3 in DCs; c-Cbl deficiency leads to RelB activation, which suppresses IL-10 transcription	RelB (ubiquitination substrate)	DCs, macrophages	Down-regulated in intestinal mucosa	*c-Cbl^f/f^CD11c^Cre/+^*	DSS; Gut fungi manipulation	Severe DSS colitis; fungi-dependent inflammation amplification	78
SCF-type Cullin-RING E3	FBW7 (FBXW7)	Defect of intestinal barrier; imbalance of intestinal immunity	Anti-inflammatory	Innate immune fate (epithelial NF-κB hyperactivation)	IEC-specific FBW7 deletion activates NF-κB pathway (↑TNFα, IL-6, IL-1β), aggravated epithelial damage, and exacerbated colitis severity	-	IECs	-	*Fbw7^ΔG^ (Vil/Cre; Fbw7^fl/fl^)*	DSS	Severe colitis	168
SCF-type Cullin-RING E3	FBW7 (FBXW7)	Defect of intestinal barrier; imbalance of intestinal immunity	Pro-inflammatory	Innate immune fate	FBW7 directly ubiquitinates EZH2, reducing CCL2/7 expression and limiting CX3CR1^hit^ macrophage recruitment	EZH2 (ubiquitination substrate, K48-linked)	Macrophages	Up-regulated in intestinal mucosa	*LysM^+^Fbxw7^fl/fl^*; AAV-shFbxw7	DSS; TNBS	Attenuated colitis (*LysM^+^Fbxw7^fl/fl^*); aggravated colitis (AAV-shFbxw7)	169
RING-like (non-classical)	Pellino1	Imbalance of intestinal immunity	Pro-inflammatory; CAC initiation-promoting	Innate immune fate (macrophage activation amplification, M2-like polarization bias)	Pellino1 ubiquitinates and stabilizes STAT3 in intestinal macrophages, enhancing STAT3 activation and amplifying macrophage-mediated inflammatory signaling to promote a pathogenic intestinal environment	STAT3 (ubiquitination substrate, K63-linked)	Macrophages	Up-regulated in colonic mucosa	*Pellino1-mKO*	DSS; AOM/DSS	Attenuated colitis; reduced CAC	52
RING-like (non-classical)	Pellino3	Imbalance of intestinal immunity	Pro-inflammatory; CAC initiation-promoting	Innate immune fate (TLR4-NF-κB/MAPK)	Pellino3 inhibits IRF4-mediated negative regulation of TLR4 signaling, thereby enhancing TLR4-driven inflammation. Loss of Pellino3 reduces colitis severity, lowers inflammation-induced colorectal tumor burden, and decreases activation of NF-κB, STAT3, and ERK pathways	IRF4 (signaling adaptor/binding partner)	Macrophages	-	*Peli3^-/-^*	DSS; AOM/DSS	Attenuated colitis; reduced CAC	53
RING-like (non-classical)	Pellino3	Imbalance of intestinal immunity	Anti-inflammatory	Innate immune fate (NF-κB/MAPK)	Pellino directly binds RIP2 via the FHA domain and catalyzes K63-linked ubiquitination through its RING-like domain; promotes TAK1/IKK recruitment, NF-κB/MAPK activation, enabling Nod2 protective signaling	RIP2 (ubiquitination substrate, K63-linked)	IECs; macrophages	Down-regulated in intestinal mucosa of CD patients	*Peli3^-/-^*	DSS; TNBS; *C. rodentium* infection	Severe colitis	122
RING-like (non-classical)	RINCK	Imbalance of intestinal immunity	Pro-inflammatory; CAC initiation-promoting	Epithelial fate (epithelial survival, barrier restitution)	IEC-specific deletion of Rinck markedly suppresses ROS production, oxidative stress, and inflammation, while overexpression of Rinck in IECs significantly exacerbates OTA/DSS-induced acute and chronic colitis	NRF2 (ubiquitination substrate, K48-linked)	IECs	Up-regulated in IBD and CRC patients	*IEC-Rinck (KO)*;* IEC-Rinck (OE)*	OTA/DSS; AOM/DSS	Attenuated colitis *(KO)*; exacerbated colitis *(OE)*	68
RING-like (non-classical)	Hakai	-	Anti-inflammatory; CAC initiation-suppressive	-	Hakai regulates FASN ubiquitination and lysosomal degradation, thereby limiting FASN-mediated lipid accumulation and linking lipid metabolism to IBD/CAC pathogenesis	FASN (ubiquitination substrate)	IECs	Up-regulated in colonic mucosa of UC and CD patients	-	DSS; AOM/DSS; IL-10-KO spontaneous colitis	-	70
RING	MARCH3	Imbalance of intestinal immunity	Anti-inflammatory; CAC initiation-suppressive	Epithelial fate (barrier homeostasis maintenance)	MARCH3 ubiquitinates IL-6Rα (K401) and gp130 (K849) to drive receptor internalization and lysosomal degradation, thereby suppressing IL-6/OSM-STAT3 pro-inflammatory signaling and limiting colitis and CAC progression	IL-6Rα (ubiquitination substrate, K48- and K63-linked); gp130 (ubiquitination substrate, K48-linked)	Macrophages	Down-regulated in CRC tissues	*March3^-/-^*	DSS; AOM/DSS	Severe colitis; aggravated CAC	72
RING	MARCH8	Imbalance of intestinal immunity	Inflammasome-suppressive	Innate immune fate	VANGL2 recruits MARCH8 to catalyze K27-linked poly-Ub of NLRP3 (K823), driving OPTN-mediated selective autophagic degradation and inhibiting NLRP3 inflammasome activation	NLRP3 (ubiquitination substrate, K27-linked)	-	-	-	-	-	65
RING	cIAP1 (Birc2), cIAP2 (Birc3)	Imbalance of intestinal immunity	Anti-inflammatory (Birc3)	Innate immune fate (NF-κB/MAPK)	cIAP1/2 act as RING E3 ligases for RIP2, mediating its K63-linked polyubiquitination and recruitment of TAK1/TAB complexes, thereby activating NOD1/2-induced MAPK (JNK, p38) and NF-κB signaling to drive cytokine and chemokine production	RIP2 (ubiquitination substrate, K63-linked)	BMDMs; HT29	-	*Birc2^-/-^; Birc3^-/-^*	DSS colitis + systemic MDP administration	Severe colitis (*Birc3^-/-^*)	83
RING	cIAP1 (Birc2)	Defect of intestinal barrier	Anti-apoptotic	Epithelial fate (TNF-induced apoptosis); Stress-adaptive fate	cIAP1 restrains TNF-induced IEC apoptosis; loss or destabilization of cIAP1 (via TWEAK signaling or Smac-mimetics) sensitizes IECs to TNF-mediated cell death and exacerbates TNF-driven enteropathies, whereas inhibition of TWEAK improves colitis	-	YAMC; MC38; macrophage	-	*Birc2^-/-^*;* Birc2^-/-^Tnfrsf1a^-/-^*	TNF-induced enteropathy model	Susceptibility to TNF-induced cell death	61
RING	Hrd1	Defect of intestinal barrier; imbalance of intestinal immunity	Anti-inflammatory; anti-apoptotic	Epithelial fate (epithelial survival and barrier maintenance)	Hrd1 activation decreases GRP78, PERK, CHOP, caspase-12, thereby limiting ER stress-induced epithelial apoptosis and inflammation	-	IECs	Down-regulated in inflamed intestinal epithelium of IBD patients	-	DSS; TNBS	Severe colitis (Hrd1 inhibitor LS102 treatment)	62
RING	RNF5	Imbalance of intestinal immunity	Anti-inflammatory; limits epithelial-derived DAMP amplification	Epithelial fate (apoptosis); Innate immune fate (DC activation threshold, NF-κB); Adaptive immune fate (Th1)	RNF5 mediates ubiquitination and proteasomal degradation of S100A8 in IECs; RNF5 deficiency leads to increased S100A8 secretion, induction of mucosal CD4⁺ T cells, Th1-mediated pro-inflammatory responses.	S100A8 (ubiquitination substrate)	IECs; dendritic cells; CD4^+^ T cells	Down-regulated in intestinal mucosa of IBD patients	*Rnf5^-/-^*	DSS	Severe colitis	56
RING	RNF8	Defect of intestinal barrier; imbalance of intestinal immunity	Anti-inflammatory	Epithelial fate (autophagy-dependent barrier maintenance)	RNF8 directly binds to AKT1 and mediates its ubiquitination, thereby suppressing AKT/mTOR signaling and restoring autophagy, and reducing pro-inflammatory cytokines	AKT1 (ubiquitination substrate)	IECs (colon epithelium); HT-29	Down-regulated in colon tissues from UC patients (GSE36807)	LV-RNF8 (RNF8 OE)	TNBS	Attenuated colitis	66
RING	RNF20	Imbalance of intestinal immunity	Anti-inflammatory; CAC initiation-suppressive	Epigenetic stability; inflammatory transcription control fate	RNF20 maintains H2Bub1 to restrain NF-κB activation; RNF20 loss reduces H2Bub1, increases p65 recruitment, decreases H3K9me3, elevates pro-inflammatory cytokine transcription	H2Bub1	MCF10A	Down-regulated in colonic mucosa of UC and CAC patients	*Rnf20^+/-^*	DSS; AOM/DSS	Susceptibility to colitis and CAC	128
RING	RNF40	Imbalance of intestinal immunity	Pro-inflammatory; CAC initiation-promoting (in intestinal epithelium)	Innate immune fate (NF-κB activation threshold)	Intestinal epithelial RNF40 sustains NF-κB signaling and promotes tumor-associated gene expression; epithelial RNF40 deletion suppresses NF-κB activity, attenuates DSS-induced colitis, and reduces tumorigenic potential	-	Colorectal cancer cell lines	-	*CAC-Cre; Rnf40^flox^*	DSS	Attenuated colitis	80
RING	RNF20/ RNF40 complex	Imbalance of intestinal immunity	Anti-inflammatory (mainly RNF20-dependent); CAC initiation-suppressive	-	The RNF20/RNF40 complex maintains H2Bub1; reduced complex activity decreases H2Bub1 and increases susceptibility to inflammation and CAC	-	-	RNF20 (but not RNF40) down-regulated in UC and CRC patients	*-*	DSS	Severe colitis	80, 128
RING	RNF31	Imbalance of intestinal immunity	Pro-inflammatory; epithelial dysfunction	Epithelial fate (barrier integrity and epithelial dysfunction)	RNF31 knockdown stabilizes NRF2, thereby enhancing oxidative stress defense, whereas NRF2 loss impairs epithelial barrier integrity and aggravates mucosal inflammation	NRF2 (ubiquitination substrate, K63-linked)	IECs	Up-regulated in UC patients	RNF31-knockdown	DSS	Attenuated colitis	69
RING	RNF31	Defect of intestinal barrier; imbalance of intestinal immunity	Pro-inflammatory	Innate immune fate (NLRP3 inflammasome activation); Epigenetic stability (tight junction loss)	RNF31 promotes NLRP3 inflammasome activation through ubiquitin-dependent regulation, thereby amplifying innate inflammatory responses and exacerbating intestinal inflammation	NLRP3 (ubiquitination substrate, K63-linked)	-	Up-regulated in UC patients	Adenovirus-mediated RNF31 knockdown	DSS	Attenuated colitis	76
RING	RNF99	Imbalance of intestinal immunity	Anti-inflammatory	Innate immune fate (macrophage inflammatory activation control)	RNF99 catalyzes ubiquitination of TAB2 at K611, promotes TAB2 proteasomal degradation, suppresses TAK1-NF-κB/MAPK signaling, and reduces pro-inflammatory cytokine production	TAB2 (ubiquitination substrate, K48-linked)	PMs, BMDMs	Down-regulated in CD14⁺ monocytes from Gram-negative-infected patients	*RNF99^-/-^*	DSS	Severe colitis	84
RING	RNF128	Imbalance of intestinal immunity	Anti-inflammatory	Immune homeostasis fate	RNF128 binds S100A8 and promotes its K63-linked ubiquitination at K36, which allows the autophagy receptor Tollip to recognize S100A8 and target it for selective autophagic degradation. Loss of RNF128 suppresses S100A8-Tollip-mediated autophagy, leading to S100A8 accumulation, enhanced macrophage cytokine production	S100A8 (ubiquitination substrate, K63-linked)	BMDMs, THP-1	Down-regulated in inflamed colonic macrophages (IBD)	*Rnf128^-/-^*	DSS; TNBS	Severe colitis	67
RING	RNF128	Imbalance of intestinal immunity; IL-6-STAT3 hyperactivation	Anti-inflammatory; CAC initiation-suppressive	Epithelial fate (proliferation restraint)	RNF128 ubiquitinates IL-6Rα and gp130 (K48-linked), promotes lysosomal degradation, suppresses IL-6-STAT3 signaling, thereby limiting epithelial hyperproliferation, inflammatory amplification and colitis-to-CAC transition	IL-6Rα and gp130 (ubiquitination substrate, K48-linked)	BMDMs, HCT116, SW620	Down-regulated in colitis and CRC tissues	*RNF128^-/-^*;* Apc^min/+^/Rnf128^+/-^*	DSS; AOM/DSS; APC^min/+^	Severe colitis; aggravated CAC	89
RING	RNF138	Colitis-to-tumor transition dysregulation	Anti-inflammatory; CAC initiation-suppressive	Innate immune fate (NF-κB); Epithelial fate (epithelial proliferation restraint)	RNF138 suppresses NF-κB activation by binding NIBP (NIK/IKKβ-binding protein) and retaining NIBP in the nucleus, thereby preventing NIBP-IKKβ cytoplasmic association and limiting p65 phosphorylation and nuclear translocation	-	HCT116, RKO	Down-regulated in CRC tissues	*RNF138^-/-^*	DSS; AOM/DSS	Severe colitis; enhanced CAC progression	85
RING	RNF180	Imbalance of intestinal immunity	Pro-inflammatory	Adaptive immune fate (Th17/Treg skewing); Epithelial fate (barrier dysfunction)	RNF180 ubiquitinates and downregulates ALKBH5, reducing ALKBH5-mediated m6A suppression on SMARCA5, leading to SMARCA5 upregulation. The increased SMARCA5 aggravates colon inflammation and induces Th17/Treg imbalance. RNF180 knockdown reverses this axis and ameliorates UC	ALKBH5 (ubiquitination substrate)	Splenic CD4⁺ T; MLN CD4⁺ T	-	sh-RNF180	DSS	Attenuated colitis	170
RING	RNF183	Epithelial stress response; mucosal barrier dysfunction	Pro-inflammatory; pro-apoptotic	Stress-adaptive fate (early epithelial stress response); Apoptotic fate (TRAIL-caspase axis)	RNF183, specifically induced in intestinal epithelial cells during early colitis, binds DR5 and mediates its K63-linked ubiquitination, promoting DR5 lysosomal trafficking and enhancing TRAIL-induced caspase-8/3 activation and epithelial apoptosis	DR5 (ubiquitination substrate, K63-linked)	IECs	Up-regulated in UC and CD inflamed colon tissues	-	DSS	-	63
RING	RNF183	Imbalance of intestinal immunity	Pro-inflammatory	Epithelial fate (barrier disruption)	RNF183 promotes intestinal inflammation by ubiquitinating and degrading IκBα, thereby activating the NF-κB pathway. RNF183 expression is negatively regulated by miR-7, which is down-regulated in IBD, leading to increased RNF183 and enhanced NF-κB-driven inflammatory responses	IκBα (ubiquitination substrate)	IECs	Up-regulated in UC and CD inflamed colon tissues	-	TNBS	-	147
RING	RNF186	ER stress-associated epithelial injury	Pro-inflammatory; pro-apoptotic	-	RNF186 localizes to ER and ubiquitinates BNip1 (K29/K63-linked), promoting BNip1 mitochondrial translocation, ER Ca²⁺ release, UPR activation (BiP, CHOP), caspase-12/-9 activation and ER stress-mediated apoptosis	BNip1 (ubiquitination substrate, K29/K63-linked)	-	-	-	-	-	91
RING	RNF186	Imbalance of intestinal immunity	Anti-inflammatory	Epithelial fate (autophagy maintenance)	RNF186 ubiquitinates EPHB2 (K892), enabling EFNB1-triggered, ULK1/PtdIns3K/ATG5-dependent autophagy in colonic epithelial cells, which promotes bacterial clearance and maintains mucosal homeostasis; loss of RNF186 impairs EPHB2-mediated autophagy	EPHB2 (ubiquitination substrate, K27-linked); EPHB3 (ubiquitination substrate, K48/K63-linked)	IECs, Ls174t, Caco2	Down-regulated in UC patients	*Rnf186^-/-^*	DSS	Susceptibility to colitis	49
RING	RNF186	Genetic susceptibility; imbalance of intestinal immunity; microbial clearance	Anti-inflammatory	Innate immune functional fate (antimicrobial response)	RNF186 ubiquitinates ATF6 at K152 to activate ATF6-UPR signaling, strengthening innate receptor responses and protecting the intestine from DSS-induced injury. RNF186 deficiency or IBD-risk variants impair ATF6 ubiquitination, reduce UPR signaling and antimicrobial immunity, leading to aggravated intestinal injury	ATF6 (ubiquitination substrate)	MDMs, BMDM, LPMs	-	RNF186 deficient (siRNA knockdown)	DSS	Severe colitis	92
RING	RNF186	Intestinal barrier dysfunction; ER stress dysregulation	Anti-inflammatory; barrier-protective	Epithelial fate (barrier homeostasis maintenance)	RNF186 mediates K48-linked ubiquitination and degradation of substrates such as occludin to maintain epithelial proteostasis. Its loss or the UC-associated A64T mutation leads to protein accumulation, elevated ER stress, increased epithelial apoptosis, impaired barrier integrity, and heightened susceptibility to intestinal inflammation	Occludin (ubiquitination substrate, K48-linked)	IECs	Down-regulated in UC patients	*Rnf186^-/-^*	DSS, Oxazolone UC-like	Severe colitis	48
RING	RNF186	Genetic susceptibility	-	-	R179X truncation disrupts RNF186 membrane localization and impairs its E3 ligase function, reducing inflammatory cytokine responses to bacterial stimuli	-	-	-	*-*	-	-	171
RING	TRAF2	Imbalance of intestinal immunity	Anti-inflammatory	Innate immune fate (macrophage inflammatory activation threshold)	TRAF2, together with TRAF3 and cIAP, promotes K48-linked ubiquitination and proteasomal degradation of c-Rel and IRF5 in macrophages, thereby limiting TLR-driven cytokine production and protecting against colitis	c-Rel/IRF5 (ubiquitination substrate, K48-linked)	Macrophages (myeloid cells)	-	*Traf2^fl/fl^lyz2^Cre/+^*	DSS	Severe colitis	79
RING	TRAF3	Imbalance of intestinal immunity	Anti-inflammatory	Innate immune fate (macrophage inflammatory programming control)	TRAF3 cooperates with TRAF2 and cIAP to promote ubiquitin-dependent degradation of the pro-inflammatory transcription factors c-Rel and IRF5 in macrophages, thereby limiting TLR/IL-1-induced pro-inflammatory cytokine production	c-Rel/IRF5 (ubiquitination substrate)	Macrophages (myeloid cells)	-	*Traf3^fl/fl^lyz2^Cre/+^*	DSS	Severe colitis	79
RING	TRAF3	-	-	-	Systemic and mucosal up-regulation (“pre-activation”) of TRAF3 in IBD patients	-	-	Up-regulated in IBD patients	*-*	-	-	172
RING	TRAF3	Defect of intestinal barrier; epithelial inflammatory signaling dysregulation	Anti-inflammatory; IL-17 signaling-restrictive	Epithelial fate (inflammatory signaling-restrictive fate, chemokine production suppression)	TRAF3 negatively regulates IL-17R signaling; NDR1 competitively disrupts TRAF3-IL-17R interaction and facilitates Act1-TRAF6 complex assembly, thereby releasing TRAF3-mediated inhibitory control	-	HeLa; mouse embryonic fibroblasts	-	*-*	-	-	173
RING	TRAF4	Mucosal immune activation	Pro-inflammatory; immune activation-promoting; disease activity-associated	Innate immune fate (UC inflammatory activity aggravation, mucosal inflammation amplification)	TRAF4 promotes immune activation by enhancing NF-κB signaling through GITR and interacting with Msn to activate the JNK pathway	-	-	Up-regulated in plasma of IBD patients	-	-	-	81
RING	TRAF5	Imbalance of intestinal immunity	Anti-inflammatory; pathogenic T cell expansion-suppressive	Adaptive immune fate (pathogenic Th cell expansion restraint fate, inflammatory cytokine production suppression)	TRAF5 restrains Th cell-mediated inflammation by limiting NF-κB activation and Th1/Th2/IFN-γ⁺IL-17A⁺ T-cell expansion	-	CD4^+^ T cells; LPMCs	-	*TRAF5^-/-^*	DSS	Severe colitis	57
RING	TRAF6	-	Pro-inflammatory; innate immune activation-promoting; inflammatory priming-associated	Innate immune fate (NF-κB/MAPK; cytokine production enhancement)	TRAF6 enhances CD40-mediated NF-κB/JNK/MAPK signaling to promote immune activation	-	-	Up-regulated in plasma, PBMCs, and inflamed colonic mucosa of IBD patients	*-*	-	-	81
RING	TRAF6	Defect of intestinal barrier; imbalance of intestinal immunity	Anti-inflammatory; microbiota-driven inflammation-restrictive; epithelial protective	-	-	-	IECs	-	*Traf6^IEC-KO^*	DSS	Exacerbated colitis	174
RING	TRIM14	Imbalance of intestinal immunity; chemokine axis dysregulation	Pro-inflammatory; CAC initiation-promoting	Innate immune fate (noncanonical NF-κB activation)	TRIM14 binds NF-κB2 p100/p52 and recruits USP14 to remove K63-linked ubiquitin chains at K332/338/341, thereby blocking p62-dependent selective autophagic degradation of p100/p52, stabilizing p100/p52, enhancing noncanonical NF-κB (RelB/p52, CXCL12/CXCL13) and promoting inflammatory responses	NF-κB2 p100/p52 (TRIM14-USP14-regulated binding partner)	PBMCs; BMDMs; BMDCs; MEFs	-	*Trim14^-/-^*	DSS; AOM/DSS	Attenuated colitis; reduced CAC	82
RING	TRIM21	Imbalance of intestinal immunity; CD4⁺ T cell-driven mucosal inflammation	Anti-inflammatory; immunosuppressive (Th1/Th17 inhibitory)	Adaptive immune fate (Th1/Th17 differentiation suppression fate, effector T-cell inflammatory program restriction)	TRIM21 inhibits TH1/TH17 differentiation in human IBD CD4^+^ T cells. TRIM21 deficiency promotes TH1/TH17 differentiation and increases IFN-γ, TNF-α, and IL-17A expression. IRF3 is a downstream target: silencing IRF3 blocks the enhanced TH1/TH17 differentiation in TRIM21^⁻/⁻^ CD4^+^ T cells	-	Naive CD4^+^ T cells	Down-regulated in intestinal mucosa of IBD patients	*Trim21^-/-^*	TNBS	Severe colitis	58
RING	TRIM21	Imbalance of intestinal immunity; imbalance of intestinal immunity	Anti-inflammatory; CAC initiation-suppressive; epithelial growth-restrictive	Epithelial fate (hyperproliferation suppression, adhesion integrity maintenance)	TRIM21 negatively regulates intestinal epithelial carcinogenesis by restraining epithelial proliferation, maintaining adhesion, limiting tissue remodeling/angiogenesis, and suppressing pro-inflammatory cytokines. Loss of TRIM21 leads to enhanced tumor-promoting inflammation and epithelial transformation	-	-	Down-regulated in intestinal mucosa of IBD and CRC patients	*Trim21^-/-^*	AOM/DSS	Severe CAC	73
RING	TRIM26	Imbalance of intestinal immunity; TLR-driven innate inflammatory amplification	Pro-inflammatory	Innate immune fate (TLR-dependent inflammatory activation fate, cytokine production enhancement)	TAB1 K11-linked polyubiquitination-dependent TAK1 activation; NF-κB/MAPK signaling amplification; pro-inflammatory cytokine induction	TAB1 (ubiquitination substrate, K11-linked)	PMs, BMDMs, MEFs, THP-1	-	*Trim26^-/-^*	DSS	Attenuated colitis	50
RING	TRIM27	Imbalance of intestinal immunity; inflammation-driven tumorigenesis promotion	Pro-inflammatory; CAC initiation-promoting	Epithelial fate; Innate immune fate (cytokine production enhancement fate)	TRIM27 recruits gp130, JAK1 and STAT3 to retromer-positive endosomal structures, facilitates JAK1-STAT3 complex assembly and STAT3 Y705 phosphorylation, amplifying IL-6 signaling cascade	-	HeLa, HT29, RKO	Up-regulated in CRC patients	*Trim27^-/^*	DSS; AOM/DSS	Attenuated colitis; reduced CAC	74
RING	TRIM27	Imbalance of intestinal immunity; NOD2 signaling dysregulation	Anti-inflammatory; NOD2 signaling-restrictive	Innate immune fate (PRRs signaling restraint fate, inflammatory cytokine production suppression)	TRIM27 directly binds NOD2 via PRY-SPRY domain and promotes K48-linked ubiquitination and proteasomal degradation of activated NOD2, thereby suppressing MDP-induced NF-κB activation	NOD2 (ubiquitination substrate, K48-linked)	HeLa	Up-regulated in CD patients	*-*	-	-	86
RING	TRIM31	Imbalance of intestinal immunity; NLRP3 inflammasome dysregulation	Inflammasome-suppressive; epithelial barrier-protective (context-dependent)	Epithelial fate (barrier integrity preservation); Innate immune fate (inflammasome suppression)	TRIM31 ubiquitinates NLRP3 (K48-linked) to promote its proteasomal degradation and restrict inflammasome activation; because NLRP3 is protective in DSS-induced mucosal injury, TRIM31 deficiency increases NLRP3 activity and IL-1β/IL-18 maturation	NLRP3 (ubiquitination substrate, K48-linked)	Macrophages; intestinal innate immune cells	-	*Trim31* ^-/-^	DSS	Attenuated colitis	51
RING	TRIM58	Imbalance of intestinal immunity	Anti-inflammatory	Innate immune fate (TLR2 signaling restraint)	TRIM58 associates with TLR2 in myeloid cells and, via its RING-dependent E3 ligase activity, promotes proteasome-dependent degradation of TLR2, thereby terminating TLR2-NF-κB/AP-1 signaling and preventing excessive IL-1β and proinflammatory cyto/chemokine production in DSS colitis	TLR2 (ubiquitination substrate)	Myeloid cells (macrophages/monocytes), IECs	Down-regulated in in UC colonic tissues	*Trim58^-/-^*;* Trim58^MC⁻/⁻^ (LysM-Cre)*	DSS	Severe colitis	87
RING	TRIM59	Imbalance of intestinal immunity; oxidative stress dysregulation	Anti-inflammatory; anti-oxidative stress; anti-apoptotic; epithelial barrier-protective	Epithelial fate (oxidative injury-resistant fate; barrier maintenance)	TRIM59 promotes KEAP1 ubiquitination and degradation, activates NRF2-driven antioxidant signaling, reduces ROS and inflammation, and consequently mitigates colitis progression	KEAP1 (ubiquitination substrate)	IECs	Down-regulated in intestinal mucosa of UC and CD patients	*IEC-KO^Trim59^*;* IEC-OE^Trim59^*	DSS	Severe colitis (*KO*); attenuated colitis (*OE*)	71
RING	TRIM62	Imbalance of intestinal immunity; antimicrobial defense-promoting	Pro-inflammatory; antimicrobial defense-promoting	Innate immune fate (CARD9-dependent inflammatory activation fate, cytokine production enhancement)	TRIM62 binds CARD9 and mediates K27-linked polyubiquitination at K125, which is essential for CARD9 activation and downstream proinflammatory cytokine production. The protective C-terminal truncated CARD9 variant fails to interact with TRIM62 and is not ubiquitinated, thereby limiting inflammatory cytokine responses	CARD9 (ubiquitination substrate, K27-linked)	-	-	*Trim62^-/-^*	DSS	Severe colitis	124
RING	TRIM33	Imbalance of intestinal immunity	Anti-inflammatory; pro-resolution	Innate immune fate (macrophage M2 polarization)	TRIM33 regulates monocyte recruitment and macrophage differentiation, and is required for the M1-to-M2 transition and adequate mTNF expression during inflammatory resolution; its loss leads to persistent inflammation	-	Monocytes, macrophages	Down-regulated in monocytes of CD patients	*Trim33^-/-^*	DSS	Severe colitis	77
RING	TRIM34	Imbalance of intestinal immunity	Anti-inflammatory; CAC initiation-suppressive	Epithelial fate (goblet cell secretory, mucus barrier maintenance)	TRIM34 in goblet cells regulates TLR signaling-induced Nox/Duox-dependent ROS synthesis, which promotes Muc2 compound exocytosis and enables proper generation of the colonic inner mucus layer. Loss of TRIM34 impairs Muc2 secretion and mucus barrier formation	-	Goblet cells	Down-regulated in colonic mucosa of UC patients	*Trim34^-/-^*	DSS; AOM/DSS	Severe colitis; enhanced CAC progression	75
RING	UHRF1	Imbalance of intestinal immune tolerance (Treg deficiency-driven inflammation)	Pro-inflammatory; immune tolerance-suppressive	Adaptive immune fate (Treg differentiation)	Uhrf1 sustains DNA methylation of Treg-related genes upon TCR stimulation, thereby limiting Treg differentiation. TGF-β induces Uhrf1 phosphorylation, cytoplasmic sequestration, and degradation, which permits Foxp3 induction and iTreg generation. Uhrf1 loss causes DNA hypomethylation and drives Treg-biased differentiation	-	CD4⁺ T cells (naive T cells → iTreg)	-	*Cd4-Cre Uhrf1^fl/fl^*	T cell transfer colitis model	Attenuated colitis	88
RING	UHRF1	Imbalance of intestinal immunity; epithelial barrier disruption	Anti-inflammatory; anti-apoptotic; barrier-protective	Epithelial fate (apoptosis); Innate immune fate (inflammatory macrophage activation fate, TNF-α hypersecretion)	Uhrf1 maintains DNA methylation at the Tnf-α promoter in macrophages, thereby restricting TNF-α production and macrophage activation. Uhrf1 deficiency or mutation causes promoter hypomethylation and excessive TNF-α expression, leading to aggravated DSS colitis. TNF-α in turn destabilizes Uhrf1 through ubiquitination-mediated degradation, creating a feed-forward activation loop	-	Macrophages	-	*Uhrf1^fl/fl^Lyz2-Cre*	DSS	Severe colitis	60

**Table 5:** Roles of deubiquitinating enzymes in intestinal inflammation and tumorigenesis.

Family	Gene	Pathogenesis of IBD/CAC	Effect	Cell fate	Mechanism/major finding	Target protein classification	Cell type	Alteration in patients	Transgenic mice	Disease model	Disease Phenotype	Reference
MJD	JOSD2	Imbalance of intestinal immunity	Anti-inflammatory; barrier-protective; CAC initiation-suppressive	Epithelial fate (apoptosis and goblet cell loss); Innate immune fate (macrophage inflammatory activation)	JOSD2 deubiquitinates K63-linked chains on IMPDH2 at K134, suppressing IMPDH2 activity and NF-κB-mediated inflammatory signaling in macrophages, thereby limiting colitis and CAC	IMPDH2 (deubiquitination substrate, K63-linked)	Macrophages (myeloid cells)	Up-regulated in colonic macrophages from UC and CD patients	*Josd2^-/-^*; AAV6-JOSD2	DSS; AOM/DSS	Severe colitis; enhanced CAC progression	108
OTU	TNFAIP3 (A20)	Defect of intestinal barrier; imbalance of intestinal immunity	Pro-inflammatory (IEC context-dependent); apoptosis-promoting	Epithelial fate (RIPK1-dependent apoptosis induction, ripoptosome enhancement, barrier breakdown)	IEC-specific A20 overexpression sensitizes epithelial cells to TNF-induced, RIPK1-dependent apoptosis by promoting Ripoptosome assembly and caspase-8/3 activation, resulting in epithelial barrier breakdown and acute inflammation	-	IECs	Up-regulated in UC and CD patients	*villin-A20 transgenic (overexpression)*	TNF injection model	Severe colitis	103
OTU	TNFAIP3 (A20)	Imbalance of intestinal immunity	Anti-inflammatory; immune homeostasis-maintaining	Innate immune fate (DC activation restraint, cytokine overproduction suppression); Adaptive immune fate (T cell activation limitation, peripheral tolerance maintenance)	A20 restricts MyD88-dependent and MyD88-independent signals in dendritic cells, limiting NF-κB activation, co-stimulatory molecule upregulation, and IL-6/TNF production, thereby preventing aberrant activation and expansion of T cells and maintaining intestinal immune homeostasis	-	Dendritic cells (CD11c⁺ DCs)	-	*A20^-/-^*,* A20^fl/fl^ Cd11c-*Cre	DSS; spontaneous colitis	Severe colitis	107
OTU	TNFAIP3 (A20)	Imbalance of intestinal immunity	Anti-inflammatory; immune homeostasis-restoring	Epithelial fate (inflammatory signaling attenuation, tissue damage reduction); Adaptive immune fate (Th17 suppression, Treg enhancement)	A20 overexpression in intestinal epithelial cells inhibits IL-6- and LPS-induced STAT3 and NF-κB activation, decreases colonic IL-17, IL-1β and TNF-α expression, and shifts the Th17/Treg balance, thereby ameliorating colitis	-	HT29	Down-regulated in colonic mucosa from UC patients	A20-overexpressing (plasmid-treated)	DSS	Attenuated colitis	121
OTU	TNFAIP3 (A20)	Defect of intestinal barrier; imbalance of intestinal immunity	Anti-inflammatory; anti-apoptotic; epithelial protective	Epithelial fate (TNF-induced apoptosis suppression, epithelial survival maintenance, barrier integrity preservation)	A20 protects intestinal epithelial cells by inhibiting TNF-induced apoptosis; A20 deficiency causes TNF-driven epithelial barrier breakdown, commensal bacterial translocation, systemic inflammation, and heightened susceptibility to colitis	-	IECs	-	*A20^IEC-KO^*	DSS	Severe colitis	104
JAMM	BRCC3	Imbalance of intestinal immunity	Pro-inflammatory; inflammasome-activating	Innate immune fate (macrophage inflammasome activation; IL-1β and IL-18 production)	BRCC3-mediated deubiquitination of NLRP3 LRR promotes NLRP3 oligomerization and inflammasome activation	NLRP3 (deubiquitination substrate, K63-linked)	Macrophages (BMDMs, THP-1-derived macrophages)	-	-	-	-	111
OTU	CYLD	Defect of intestinal barrier; imbalance of intestinal immunity	Pro-inflammatory (context-dependent); necroptosis-promoting	Epithelial fate (RIP3-mediated necroptosis induction, Paneth cell loss, barrier disruption)	CYLD deubiquitinase activity promotes RIP1/RIP3-dependent necroptosis downstream of TNFR1; inhibition of CYLD catalytic activity in IECs (CYLDΔ932^IEC^) prevents RIP3-mediated epithelial necrosis and spontaneous colitis in FADD^IEC-KO^ mice	-	IECs	-	CYLDΔ932^IEC^, FADD^IEC-KO^	Spontaneous microbiota-dependent colitis in FADD^IEC-KO^ mice	Attenuated colitis	175
OTU	CYLD	Imbalance of intestinal immunity	Anti-inflammatory; CAC initiation-suppressive	Epithelial fate (inflammation-driven survival signaling enhancement); Innate immune fate (NF-κB/JNK hyperactivation, cytokine overproduction)	CYLD removes K63-linked ubiquitin from TRAF2 and NEMO to restrain NF-κB/JNK signaling. CYLD loss leads to TRAF2/NEMO hyperubiquitination, sustained NF-κB/JNK activation, and increased CAC	TRAF2 and NEMO (deubiquitination substrate, K63-linked)	B cells, T cells, macrophages	-	*Cyld^-/-^*	DSS; DSS-induced CAC	Severe colitis; prone to CAC	109
OTU	CYLD	Defect of intestinal barrier; imbalance of intestinal immunity	Anti-inflammatory; inflammasome-suppressive	Epithelial fate (barrier integrity maintenance, microbial translocation limitation); Innate immune fate (inflammasome activation restraint, IL-18 maturation control)	CYLD deubiquitinates NLRP6 to restrict K63-linked ubiquitination, limiting NLRP6-ASC complex formation, caspase-1 activation, and IL-18 maturation, thereby preventing excessive intestinal inflammation	NLRP6 (deubiquitination substrate, K63-linked)	IECs	-	*Cyld^-/-^*; *IEC-Cyld*^Δ9^	*C. rodentium-*induced colitis; TNBS	Severe colitis	105
OTU	CYLD	Imbalance of intestinal immunity	Anti-inflammatory; innate immune signaling-terminating	Innate immune fate (TLR-NF-κB/MAPK termination, cytokine overproduction suppression)	CYLD deubiquitinates TRAF6 to terminate TLR-triggered NF-κB signaling; in this study GIT2 recruits CYLD to TRAF6 and enhances CYLD-mediated deubiquitination of TRAF6, thereby limiting TLR-induced NF-κB and MAPK activation	TRAF6 and NEMO (deubiquitination substrate, K63-linked)	BMDMs	-	-	-	-	125
OTU	CYLD (short splice variant, sCYLD)	Imbalance of intestinal immunity	Pro-inflammatory; immune tolerance-disruptive; TGF-β signaling-suppressive	Adaptive immune fate (Treg/Th17 differentiation inhibition, Th1 polarization enhancement, effector memory T cell expansion)	sCYLD promotes K63-linked ubiquitination and nuclear translocation of SMAD7 in CD4⁺ T cells, recruiting SMAD7 into SMAD3/4 DNA-binding complexes to inhibit TGF-β signaling, thereby impairing Treg and Th17 differentiation, enhancing Th1 effector responses, and driving colitis	SMAD7 (deubiquitination substrate, K63-linked)	CD4⁺ T cells	Up-regulated in colonic lamina propria T cells from CD patients.	*sCYLD/SMAD7*	Spontaneous colitis	Severe colitis	120
OTU	OTUD1	Imbalance of intestinal immunity	Anti-inflammatory; barrier-protective	Innate immune fate (macrophage inflammatory activation; NF-κB-dependent TNF-α/IL-6/IL-1β production)	OTUD1 binds RIPK1 and removes K63-linked ubiquitin chains (notably at K627), blocking NEMO recruitment and RIPK1-mediated NF-κB activation, thereby limiting proinflammatory cytokine production	RIPK1 (deubiquitination substrate, K63-linked)	Hematopoietic cells	Down-regulated in intestinal mucosa from UC patients	*Otud1^-/-^*	DSS	Susceptibility to colitis	126
OTU	OTUD5	Imbalance of intestinal immunity	Pro-inflammatory	Innate immune fate (APC/macrophage inflammatory activation; TNF-α production); Adaptive immune fate (Th17 differentiation regulation; context-dependent)	OTUD5 is induced by IFN-γ via p38/MAPK in intestinal lamina propria antigen-presenting cells and sustains the inflammatory cytokine response, as antisense-mediated OTUD5 knockdown in IBD and TNBS-colitis LPMCs reduces p38 activation and TNF-α expression	-	Lamina propria antigen-presenting cells and epithelial cells	Up-regulated in inflamed ileal and colonic mucosa from UC and CD patients	-	TNBS	-	112
OTU	OTUD6A	Imbalance of intestinal immunity	Pro-inflammatory; inflammasome-activating; CAC initiation-promoting	Innate immune fate (macrophage NLRP3 inflammasome activation; IL-1β/IL-18 maturation and pyroptosis)	OTUD6A binds NLRP3 and removes K48-linked ubiquitin chains at K430 and K689, stabilizing NLRP3 and enhancing NLRP3 inflammasome activation and IL-1β production in macrophages	NLRP3 (deubiquitination substrate, K48-linked)	Macrophages (BMDMs; myeloid cells)	Up-regulated in colonic mucosa from UC patients	*Otud6a* ^-/-^	DSS; TNBS; AOM/DSS	Attenuated colitis; reduced CAC	113
OTU	OTULIN	Defect of intestinal barrier; imbalance of intestinal immunity	Anti-inflammatory; anti-apoptotic; barrier-protective	Epithelial fate (TNF-induced apoptosis, epithelial destruction, barrier disruption)	OTULIN deficiency impairs TNFR1 complex I formation and LUBAC recruitment upon TNF stimulation, promotes formation of cytosolic death-inducing complex II (FADD-caspase-8), leading to excessive TNF-induced epithelial apoptosis and barrier breakdown	-	IECs	-	*OTULIN^IEC^* ^-KO^	DSS	Susceptibility to colitis	97
USP	USP1	Genetic susceptibility	-	-	SNP (rs1748195) in USP1 gene is associated with CD risk		-	-	-	-	-	176
USP	USP3	Genetic susceptibility	-	-	Polymorphisms in USP3 genes are associated with both CD and UC patients		-	-	-	-	-	45
USP	USP4, USP40	Genetic susceptibility	-	-	Polymorphisms in USP4 and USP40 genes are associated with CD and UC patients		-	-	-	-	-	21, 45, 177
USP	USP5, USP15, USP19, USP39	Genetic susceptibility	-	-	Polymorphisms in USP5, 15, 18, 39 genes are associated with UC patients		-	-	-	-	-	177
USP	USP7	Defect of intestinal barrier; imbalance of intestinal immunity	Pro-inflammatory; pro-oxidative	Epithelial fate (barrier disruption, epithelial injury	USP7 deubiquitinates and stabilizes AMBRA1, which suppresses NRF2 antioxidant signaling and increases oxidative stress in IECs; USP7 inhibition reduces AMBRA1, restores NRF2 activity, and alleviates colitis	AMBRA1 (deubiquitination substrate, K48-linked)	IECs	-	USP7 inhibitor (P5091)	DSS	Attenuated colitis	102
USP	USP7	Imbalance of intestinal immunity; impaired Treg stability	Anti-inflammatory; maintains Treg suppressive function	Adaptive immune fate (Foxp3 stabilization, Treg suppressive function maintenance)	USP7 deubiquitinates and stabilizes Foxp3, enhancing Treg suppressive function; USP7 inhibition or knockdown reduces Foxp3 and abolishes Treg-mediated resolution of colitis	Foxp3 (deubiquitination substrate)	Treg cells	-	Foxp3-GFP mice; Treg with shUSP7	Adoptive-transfer colitis	-	116
USP	USP8	Imbalance of intestinal immunity	Anti-inflammatory; immune homeostasis-maintaining	Adaptive immune fate (thymocyte maturation, IL-7Rα expression, Treg suppressive function)	USP8 acts in the TCR signalosome and supports Foxo1-dependent IL-7Rα expression, maintaining T-cell development, homeostasis and Treg function; its loss disrupts these programs and permits expansion of colitogenic γδ T cells	Gads and 14-3-3β (deubiquitination substrate)	Thymocytes and peripheral T cells	-	*Usp8^f/f^Cd4-*Cre; *Usp8^f/f^Cd4-*CreERT2	Spontaneous colitis	Severe colitis	117
USP	USP9X	Defect of intestinal barrier	Anti-inflammatory; CAC initiation-suppressive	Epithelial fate (crypt progenitor proliferation control, goblet/Paneth cell differentiation, epithelial regeneration)	USP9X binds FBW7α/β and removes degradative K48-linked polyubiquitin chains, stabilizing FBW7 and thereby promoting degradation of SCF(FBW7) substrates (c-MYC, NICD1, c-JUN, cyclin E); intestinal Usp9x loss lowers Fbw7, increases these oncoproteins	FBW7 (deubiquitination substrate, K48-linked)	IECs	-	*Usp9x^fl/fl^Villin-Cre*	DSS; AOM/DSS	Severe colitis; prone to CAC	98
USP	USP13	Defect of intestinal barrier; ER stress dysregulation	Anti-inflammatory; anti-apoptotic; stress-adaptive; barrier-protective	Epithelial fate (IEC apoptosis inhibition, barrier integrity maintenance)	USP13 in intestinal epithelial cells binds GRP78 and removes K63-linked ubiquitin at K327 via its catalytic C343 site, suppressing GRP78-mediated ER stress and apoptosis and maintaining tight-junction-dependent barrier integrity during DSS colitis	GRP78 (deubiquitination substrate, K63-linked)	IECs	Down-regulated in inflamed colonic/rectal mucosa of IBD patients	*Usp13^IEC^*^-KO^; AAV-Usp13	DSS	Severe colitis (*Usp13^IEC^*^-KO^); attenuated colitis (AAV-Usp13)	25
USP	USP16	Imbalance of intestinal immunity	Pro-inflammatory; CAC initiation-promoting	Innate immune fate (macrophage activation, cytokine and chemokine production)	USP16 directly binds IKKβ and selectively removes K33-linked polyubiquitin chains from IKKβ at lysine 238, facilitating IKKβ interaction with p105, enhancing p105 phosphorylation and processing to p50, thereby selectively amplifying canonical NF-κB signaling without affecting IκBα phosphorylation	IKKβ (deubiquitination substrate, K33-linked)	Macrophages; BMDMs; myeloid cells	Up-regulated in colonic macrophages and inflamed mucosa of UC and CD patients	*Usp16^fl/fl^Lyz2-Cre^+^*	DSS; AOM/DSS	Attenuated colitis; reduced CAC	114
USP	USP21	Imbalance of intestinal immunity	Anti-inflammatory (via Th17 suppression); immune differentiation-restrictive	Adaptive immune fate (Th17 differentiation inhibition, CD4⁺ T cell lineage regulation)	USP21 interacts with AhR and removes K48-linked polyubiquitin chains at K432, stabilizing AhR but suppressing its transcriptional activity. Through this deubiquitination-dependent inhibition of AhR, USP21 negatively regulates Th17 differentiation	AhR (deubiquitination substrate, K48-linked)	CD4^+^ T cell; Th17 cells	-	-	-	-	118
USP	USP22	Defect of intestinal barrier; imbalance of intestinal immunity	Anti-inflammatory; CAC initiation-suppressive; epigenetic transcription-repressive	Epithelial fate (epithelial damage aggravation, barrier impairment); Innate immune fate (cytokine overproduction, immune cell infiltration)	USP22 epigenetically suppresses SPARC transcription by removing monoubiquitinated H2B (H2Bub1) and reducing H3K27ac occupancy at the SPARC promoter and gene body, thereby limiting SPARC expression and restraining inflammation-associated gene programs	H2Bub1 (deubiquitination substrate)	IECs; HCT116	Down-regulated in UC patients with neoplasia	*Usp22^fl/fl^ Villin-CreER^T2^*	DSS; DSS (*Apc*^1638N/+^ CAC model)	Severe colitis; prone to CAC	99
USP	USP25	Defect of intestinal barrier; imbalance of intestinal immunity	Anti-inflammatory; barrier-protective	Epithelial fate (tight junction maintenance, epithelial regeneration, barrier integrity preservation)	USP25 interacts with phospho-STAT3 and catalyzes K48-linked deubiquitination of p-STAT3^Y705^ at K409, preventing its proteasomal degradation and maintaining STAT3 activity to promote IL-10/IL-22 and tight junction protein expression in intestinal epithelial cells	STAT3 (deubiquitination substrate, K48-linked)	IECs	Down-regulated in colonic mucosa of UC patients	*Usp25^-/-^*; AAV8-Usp25	DSS	Severe colitis	100
USP	USP25	Genetic susceptibility	-	-	USP25 locus shows genome-wide significant association with IBD in African Americans; rs7278277 near USP25 is a GWS SNP for IBD	-	-	-	-	-	-	24
USP	USP25	Defect of intestinal barrier; imbalance of intestinal immunity	Pro-inflammatory (infection context); epithelial proliferative signaling-promoting; CAC initiation-promoting	Epithelial fate (secretory lineage regulation, proliferative signaling enhancement); Innate immune fate (suppressed antibacterial responses, cytokine remodeling)	USP25 deubiquitinates and stabilizes TRAF3 to restrain TLR-triggered signaling; in the intestine, USP25 in non-hematopoietic cells limits antibacterial cytokines/antimicrobial peptides and, by promoting Wnt signaling and restraining SOCS3-pSTAT3, supports colitis-associated tumorigenesis, whereas USP25 deficiency or inhibition enhances antibacterial immunity	-	ECs; LPMCs; secretory cells	-	*Usp25^-/-^*	DSS; AOM/DSS	Attenuated colitis; reduced CAC	115
USP	USP28	Imbalance of intestinal immunity	Anti-inflammatory; immune homeostasis-maintaining	Adaptive immune fate (T cell activation control, Th17 differentiation modulation, enhanced Treg suppressive function, IL22 overproduction)	USP28 in T cells restrains STAT5 phosphorylation and IL-22 production; Usp28*^-/-^* T cells show enhanced STAT5 activation, increased IL-22/IFN-γ and altered Th17/Treg functions, leading to greater susceptibility to DSS-induced colitis	-	T lymphocytes (CD4⁺ Th17/Treg/iTreg and CD8⁺ T cells)	-	*Usp28^-/-^*	DSS (acute/chronic)	Severe colitis	119
USP	USP38	Imbalance of intestinal immunity	Anti-inflammatory; transcription-repressive	Innate immune fate (macrophage/DC inflammatory activation, IL-6 and IL-23a overproduction)	USP38 deubiquitinates H2B K120 and recruits/stabilizes KDM5B to remove H3K4me3 at Il6 and Il23a promoters, thereby limiting NF-κB (p65, c-Rel, p50) binding and selectively suppressing IL-6 and IL-23α expression	KDM5B (deubiquitination substrate, K48-linked)	Macrophages (BMDMs, peritoneal macrophages); BMDCs	-	*Usp38^-/-^*	DSS	Severe colitis	110
USP	USP44	Genetic susceptibility	-	-	USP44 promoter hypermethylation is part of a 5-gene methylation panel that is enriched in neoplastic and non-neoplastic colonic mucosa from IBD patients with dysplasia/cancer and from high-risk IBD patients, serving as an early biomarker of IBD-associated colorectal neoplasia	-	Colonic mucosal biopsies	Hypermethylated in IBD mucosa	-	-	-	178
USP	USP47	Defect of intestinal barrier; imbalance of intestinal immunity	Anti-inflammatory; epithelial protective	Epithelial fate (NF-κB activation control, epithelial injury attenuation)	USP47 binds TRAF6 in intestinal epithelial cells and removes K63-linked ubiquitin chains (notably at Lys124), thereby restraining TRAF6-dependent NF-κB activation and epithelial inflammatory cytokine production and protecting against colitis	TRAF6 (deubiquitination substrate, K63-linked)	IECs	Down-regulated in colonic mucosa of UC and CD patients	*Usp47^-/-^*	DSS	Severe colitis	101

## Abbreviations

AOM: azoxymethane
ASC: apoptosis-associated speck-like protein containing a CARD
ATP: adenosine triphosphate
CAC: colitis-associated cancer
CD: Crohn's disease
CD4⁺: cluster of differentiation 4-positive
CHOP: C/EBP homologous protein
CLRs: C-type lectin receptors
CRC: colorectal cancer
CRISPR: clustered regularly interspaced short palindromic repeats
DCs: dendritic cells
DSS: dextran sulfate sodium
DUBs: deubiquitinating enzymes
E1: ubiquitin-activating enzyme
E2: ubiquitin-conjugating enzyme
E3: ubiquitin ligase
EMT: epithelial-mesenchymal transition
ER: endoplasmic reticulum
ERK1/2: extracellular signal-regulated kinase 1/2
GRP78: glucose-regulated protein 78
GWAS: genome-wide association studies
IBD: inflammatory bowel disease
IECs: intestinal epithelial cells
IGF2BP2: insulin-like growth factor 2 mRNA-binding protein 2
IKKβ: inhibitor of nuclear factor kappa-B kinase subunit beta
IL: interleukin
IRAKs: interleukin-1 receptor-associated kinases
IRF: interferon regulatory factor
JAMM: JAB1/MPN/Mov34 metalloenzyme
JNK: c-Jun N-terminal kinase
KDM5B: lysine demethylase 5B
KEAP1: Kelch-like ECH-associated protein 1
lncRNAs: long non-coding RNAs
LPS: lipopolysaccharide
MAPK: mitogen-activated protein kinase
miRNAs: microRNAs
MJD: Machado-Joseph disease
mRNA: messenger RNA
MyD88: myeloid differentiation primary response 88
ncRNA: non-coding RNA
NF-κB: nuclear factor kappa-B
NLRP3: NOD-like receptor family pyrin domain containing 3
NLRP6: NOD-like receptor family pyrin domain containing 6
NOD2: nucleotide-binding oligomerization domain-containing protein 2
NRF2: nuclear factor erythroid 2-related factor 2
OTU: ovarian tumor
PERK: protein kinase RNA-like endoplasmic reticulum kinase
PINK1: PTEN-induced kinase 1
PPARγ: peroxisome proliferator-activated receptor gamma
PROTAC: proteolysis-targeting chimera
PRRs: pattern recognition receptors
RelA: RELA proto-oncogene, NF-κB subunit
RIP2: receptor-interacting serine/threonine kinase 2
RIPK1: receptor-interacting protein kinase 1
ROS: reactive oxygen species
scRNA-seq: single-cell RNA sequencing
STAT3: signal transducer and activator of transcription 3
TAB: TAK1-binding protein
TAK1: transforming growth factor-beta-activated kinase 1
TGF-β: transforming growth factor-beta
Th1: T helper 1
Th17: T helper 17
TLR4: Toll-like receptor 4
TLRs: Toll-like receptors
TNBS: 2,4,6-trinitrobenzene sulfonic acid
TNF-α: tumor necrosis factor-alpha
TNFR1: tumor necrosis factor receptor 1
TNFRs: tumor necrosis factor receptors
Ub: ubiquitin
UBC9: ubiquitin-conjugating enzyme 9
UC: ulcerative colitis
UCHL5: ubiquitin C-terminal hydrolase L5
UMEs: ubiquitin-modifying enzymes
UPR: unfolded protein response
USP: ubiquitin-specific protease
VDR: vitamin D receptor
